# Supplementary material for: Coupling cellular drug-target engagement to downstream pharmacology with CeTEAM
Source: Nat Commun. 2024 Dec 6;15:10347. doi: 10.1038/s41467-024-54415-7 (PMC11624193; doi:10.1038/s41467-024-54415-7)
Supplement: Supplementary file 1 — Supplementary Information [file 41467_2024_54415_MOESM1_ESM.pdf]

Supplementary Information

Coupling cellular drug-target engagement to downstream pharmacology with CeTEAM

Nicholas C.K. Valerie, Kumar Sanjiv, Oliver Mortusewicz, Si Min Zhang, Seher Alam, Maria J. Pires, Hannah Stigsdotter, Azita Rasti, Marie-France Langelier, Daniel Rehling, Adam Throup, Oryn Purewal-Sidhu, Matthieu Desroses, Jacob Onireti, Prasad Wakchaure, Ingrid Almlöf, Johan Boström, Luka Bevc, Giorgia Benzi, Pål Stenmark, John M. Pascal, Thomas Helleday, Brent D.G. Page, and Mikael Altun

Correspondence to: nicholas.valerie@ki.se

Supplementary Discussion

PARP1 L713F-GFP accumulation following proteasome and/or autophagosome inhibition

Notably, PARP1 L713F-GFP appeared to demonstrate negligible accumulation following proteasome inhibition and detection by western blotting (**Supplementary Fig. 1g**) or live cell fluorescent microscopy (**Supplementary Fig. 1h**). Further, autophagy inhibition by bafilomycin A1 (specifically, inhibition of autophagosome-lysosome fusion) or their combination also did not affect global PARP1 L713F-GFP signal when monitored by microscopy (**Supplementary Fig. 1h**). PARP1 L713F-nLuc, however, was partially rescued by MG-132 treatment over 6 hours as seen by western blot (**Supplementary Fig. 16b**). Upon closer inspection, it became clear that proteasomal inhibition via MG-132 treatment resulted in specific accumulation of L713F-GFP in the nucleoli (**Supplementary Fig. 1h, and Supplementary Fig. 14c**). It has long been known that significant fractions of PARP1 reside in the nucleoli[1], but it has only recently emerged that, in addition to acting as a reservoir for the DDR, PARP1 is a key regulator of nucleolar biology[2]. Thus, this selective accumulation of L713F-GFP may relate to PARP1-associated functions in the nucleolus and will require further investigation.

Congruence of PARP1 L713F with PARP1 biology

PARP1 is equally amenable to GFP fusions on the N- or C-terminus without compromising functionality[3], so differential tagging of WT vs L713F PARP1 should not affect interpretation of DDR responsiveness between the two variants. Indeed, both variants were recruited to laser microirradiation-induced DNA damage sites, although L713F-GFP had slightly attenuated kinetics, as previously reported (**Supplementary Fig. 12d and e**)[4]. FRAP analyses also suggested that L713F-GFP transient nuclear mobility was slightly worse (**Supplementary Fig. 12f-j**). PARP1 WT (heterogeneous population) and L713F (clonal population) were then profiled longer-term for recruitment to damage sites and proximal markers of PARP1 activity with PARPi pre-treatment for one or 24 hours (**Supplementary Fig. 13a**, **b**, **and h**). While the mean fluorescence intensity of WT PARP1 was unaffected by PARPi alone, it temporally increased following DNA damage and then regressed to baseline (**Supplementary Fig. 13c**). However, one-hour pre-treatment with olaparib (5 μM) or talazoparib (0.5 μM) appeared to sustain higher GFP signals 60 minutes post-radiation (**Supplementary Fig. 13c and f**)[5]. PARP1 L713F mimicked WT GFP

intensity in the absence of PARPi but was notably increased following one hour of PARPi treatment (**Supplementary Fig. 13b and c**). Nuclear PAR and γH2A.X trends following DNA damage were similar between PARP1 WT and L713F-expressing cells, *i.e.* PARPi suppressed PAR formation but did not affect γH2A.X (**Supplementary Fig. 13d and e**). Notably, however, basal PAR and γH2A.X levels were significantly elevated in L713F-GFP-expressing cells (**Supplementary Fig. 13b, d, and e**). PARP1 L713F-GFP remained high after 24 hours of PARPi, but both PAR and γH2A.X levels had risen even prior to DNA damage induction, which was only reciprocated in talazoparib-treated WT PARP1 cells (**Supplementary Fig. 13h-k**). This indicated that PARP trapping in the unperturbed, cycling cells had become predominant. Collectively, these data suggested that PARP1 L713F-GFP behaves similarly to PARP1 WT following PARPi treatment in the absence or presence of DNA damage.

Reintroduction of PARP1 L713F-GFP into PARP1 KO HeLa cells was previously reported to be toxic via NAD+ exhaustion due to continual, DNA-independent PAR formation[4]. For this reason, we utilized a doxycycline-inducible promoter in initial experiments to minimize any potential toxicity. When L713F-GFP was introduced into U-2 OS or HCT116 cells, which contain both endogenous alleles of WT PARP1, we were able to confirm elevated basal PAR and γH2A.X in comparison to WT PARP1 (**Supplementary Fig. 13d and e**) but saw no overt signs of toxicity or stress over several days/weeks of culture (**Supplementary Fig. 13g**). Nonetheless, the elevated PAR and γH2A.X levels were suppressed by PARPi treatment, as before[4] (**Supplementary Fig. 13b, d, e**). This discrepancy may reflect differences in host cell background, where the HeLa PARP1 KO cells may have physiologically adapted to PARP1 inactivation and are unable to cope with the acute, constitutive activity of PARP1 L713F. A related possibility concerns the differences in heterologous expression systems. Specifically, transient transfections can result in uneven transgene expression, where very high expression of PARP1 L713F in a fraction of target cells could become problematic for viability. The use of lentiviral transduction, where we observe better uniformity and levels of transgene expression at or below endogenous protein abundances, has likely mitigated these concerns in our experimental system.

Supplementary Table 1. Data collection and refinement statistics of the NUDT15-NSC56456 co-crystal structure.

	NUDT15 + NSC56456<br>PDB ID 7NR6
**Data collection**	
Beamline	BESSY 14.1
Wavelength (Å)	0.9184
Space group	$P\ 2_1\ 2_1\ 2_1$
Cell dimensions	
a, b, c (Å)	46.84, 49.03, 135.38
α, β, γ (°)	90, 90, 90
Resolution (Å)	67.69-1.80(1.84-1.80)*
R_{merge}	7.9 (91.9)*
CC1/2	0.998 (0.757)*
$\langle I / \sigma I \rangle$	12.7 (1.9)*
Total observations	199,093 (12,046)*
Unique observations	29,634 (1,714)*
Completeness (%)	99.7 (99.7)*
Redundancy	6.7 (7.0)*
**Refinement**	
R_{work} / R_{free}	18.7 / 20.6
No. atoms	
Protein	2502
Ligand/ion	44
Water	284
Average B-factors ($Å^2$)	
Protein	28.8
Ligand/ion	27.9
Water	38.5
R.m.s. deviations	
Bond lengths (Å)	0.006
Bond angles (°)	0.82
Ramachandran statistics	
Favoured (%)	98.7
Allowed (%)	1.3
Outliers (%)	0

A single crystal was used for data collection.
*Values in parentheses are for highest-resolution shell

Supplementary Table 2. Small molecule screening details

Category	Parameter	Description
Assay	Type of assay	Cell-based, dual luminescence assay
	Target	PARP1 L713F (human PARP1)
	Primary measurement	Abundance of PARP1 L713F-nLuc relative to an akaLuc normalization signal
	Key reagents	• HCT116 colon carcinoma cells expressing pCW57.1-PARP1 L713F-nLuc and pLenti CMV Blast-akaLuc (see Methods)<br>• akaLumine HCl (TokeOni; Sigma Aldrich)<br>• Furimazine (Promega Nano-Glo Assay Kit)
	Assay protocol	See Methods section
	Additional comments	Luminescence readout is from intact, live cells
Library	Library size	1187
	Library composition	MedChemExpress Epigenetics & Selleck Nordic Oncology drug-like libraries offered by the SciLifeLab Compound Center
	Source	The libraries were spotted from up to 10 mM DMSO solutions in Labcyte 384 LDV plates using an Echo 550
	Additional comments	SciLifeLab Compound Center spotted compounds in assay plates.
Screen	Format	96-well format<br>Assay plate: Greiner Cell Culture Microplate, 96 Well, PS, F-Bottom (Chimney Well), White, Cellstar® Tc, Lid With Condensation Rings, Sterile (catalog # 655083)
	Concentration(s) tested	Compound concentration at 10 µM, DMSO concentration at 0.1%
	Plate controls	Negative control: Test cells with 0.75 µg/µL doxycycline (DOX) added + 0.1% DMSO [v/v] (Column 1 on each plate)<br>Positive control: Test cells + DOX treated with 10 µM veliparib (0.1% DMSO [v/v]; Column 2 on each plate)
	Reagent/ compound dispensing system	Compound dispensing system: Echo 550 from Labcyte<br>Cell dispensing system: Multidrop Combi from Thermo Scientific<br>Luminescence reagent dispensing: Eppendorf dispensing multichannel pipet
	Detection instrument and software	CLARIOStar microplate reader and analysis software (BMG LABTECH)
	Assay validation/QC	• Negative control – average nLuc/akaLuc ratio: 0.014, standard deviation: 0.004; positive control – average nLuc/akaLuc: 0.077, standard deviation: 0.018; average Z' factor/plate: 0.29.<br>• To account for extreme viability and expression variabilities, compounds with akaLuc signals deviating >4 SDs from control means on each plate were excluded from analysis.<br>• QC also included monitoring of plate edge effects and hit distribution of the hits, with no additional corrections necessary.
	Correction factors	Exclusion of compounds resulting in akaLuc signals >4 SDs from controls per plate.
	Normalization	L713F-nLuc signal is normalized to the respective akaLuc signal in each well (nLuc/akaLuc ratio). For simplified data visualization, the nLuc/akaLuc ratio was set relative to the mean for the negative control (+DOX, DMSO) to generate fold-change.
	Additional comments	None
Post-HTS analysis	Hit criteria	Hit threshold: Average $\log_2$(nLuc/akaLuc) ratio of test samples with akaLuc <4 SDs (-0.0148) ± 2xSD = -1.5501 (negative) or 1.4304 (positive). Hits were also defined as +3xSDs = 2.2881.
	Hit rate	6.31% (53/840)
	Additional assay(s)	Hit confirmation was performed with the same assay conditions (10 µM final concentration) in triplicate with 1-2 sets (total of up to 6 replicates)
	Confirmation of hit purity and structure	Hits confirmed by LCMS
	Additional comments	None

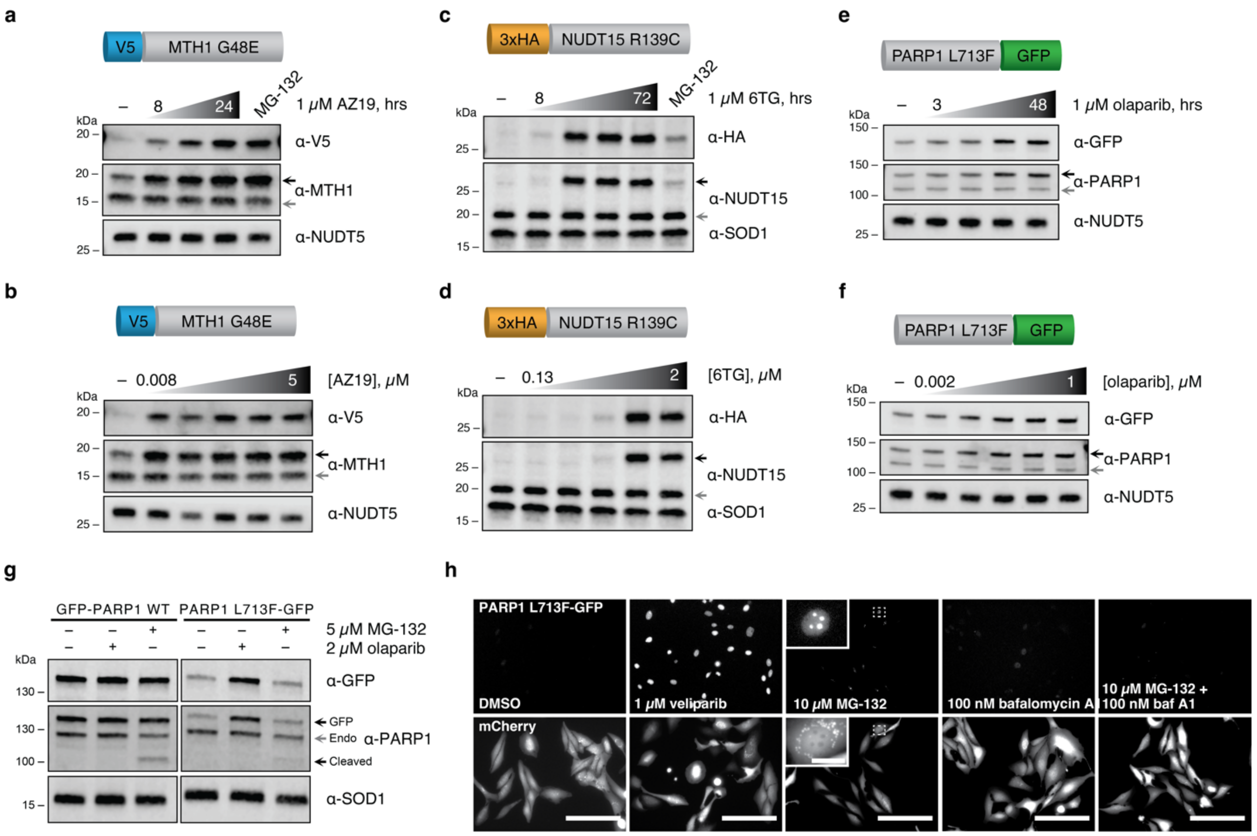

Supplementary Figure 1. Initial time and dose characterization of MTH1, NUDT15, and PARP1 drug biosensors. a, Representative blot (n=2) of U-2 OS V5-MTH1 G48E cells incubated with DMSO (24 hours), 5 µM MG-132 (6 hours), or 1 µM AZ19 (8-24 hours). **b**, Representative blot (n=2) of U-2 OS V5-MTH1 G48E cells incubated with DMSO or AZ19 (0.008-5 µM) for 24 hours. **c**, Representative blot (n=2) of HCT116 3-6 3xHA-NUDT15 R139C cells incubated with DMSO (72 hours), 5 µM MG-132 (6 hours), or 1 µM 6TG (8-72 hours). **d**, Representative blot (n=2) of HCT116 3-6 3xHA-NUDT15 R139C cells incubated with DMSO or 6TG (0.13-2 µM for 72 hours. **e**, Representative blot (n=2) of U-2 OS PARP1 L713F-GFP cells incubated with DMSO (48 hours) or 1 µM olaparib (3-48 hours). **f**, Representative blot (n=2) of U-2 OS PARP1 L713F-GFP cells incubated with DMSO or olaparib (0.0016-1 µM) for 48 hours. **g**, Representative blot (n=2) of U-2 OS GFP-PARP1 WT or L713F-GFP cells incubated with DMSO, 2 µM olaparib (24 hours), or 5 µM MG-132 (6 hours). Black arrow – exogenous fusion protein; grey arrow – endogenous protein. **h**, U-2 OS PARP1 L713F-GFP/mCherry cells were incubated with DMSO, 1 µM veliparib (24 hours), 10 µM MG-132, 100 nM bafilomycin A1, or 10 µM MG-132 and 100 nM bafilomycin A1 (each for 6 hours) before live cell fluorescence microscopy. Scale bar: 200 µm, 20 µm for inset. In all cases, cells were induced with doxycycline before drug addition.

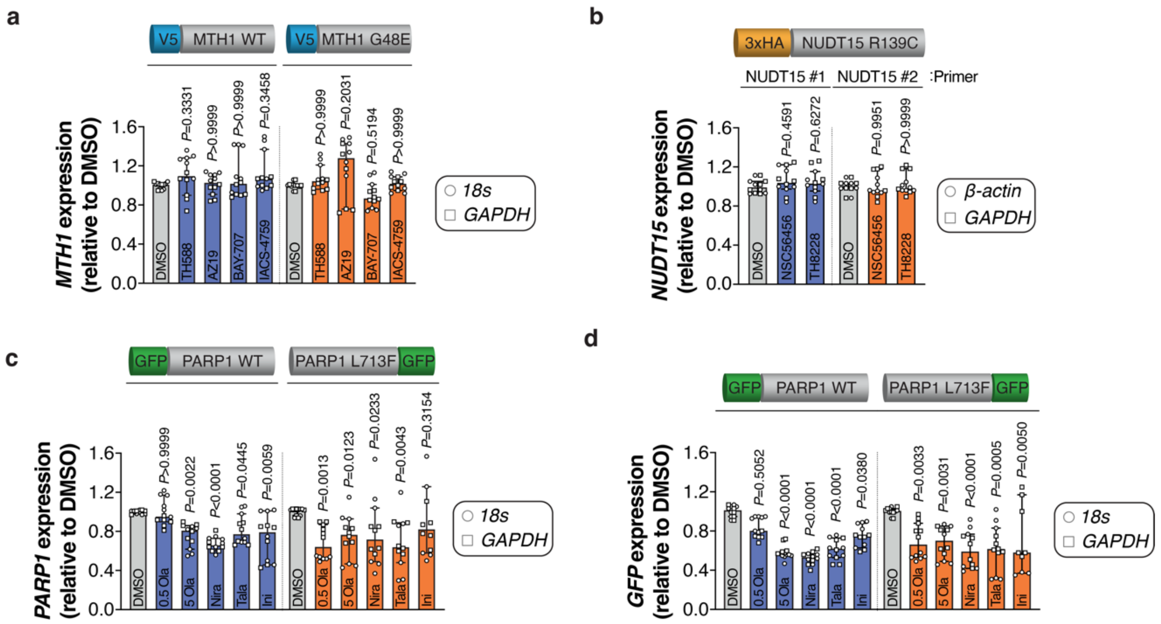

Supplementary Figure 2. Drug biosensor responses are unrelated to expression changes. a, U-2 OS V5-MTH1 WT or G48E cells were treated with DMSO, 2.5 µM TH588, 1 µM AZ19, 1 µM BAY-707, or 1 µM IACS-4759 for 24 hours before RT-qPCR analysis on total *MTH1* expression. **b**, HCT116 3-6 3xHA-NUDT15 R139C cells were treated with DMSO, 20 µM NSC56456, or 20 µM TH8228 for 24 hours before RT-qPCR analysis on total *NUDT15* expression. **c**, U-2 OS GFP-PARP1 WT or PARP1 L713F-GFP cells were treated with DMSO, 0.5 or 5 µM olaparib, 5 µM niraparib, 0.5 µM talazoparib, or 10 µM iniparib for 24 hours before RT-qPCR analysis. Total *PARP1* transcripts or *GFP* transcripts (**d**) were quantified. For all, median values from n=4 run in triplicate (except for $n_{GFP,\mathrm{L713F,iniparib}}$=3) and normalized to each housekeeping gene with 95% confidence intervals plotted (*18s* – circles, *β-actin* – circles, *GAPDH* – squares). DMSO (grey) and drug-treated samples (blue [WT] or orange [mutant]) indicated. *P* values shown from Kruskal-Wallis test with multiple comparisons to DMSO controls for each primer or cell set (Dunn's test; Kruskal-Wallis statistic = $4.457_{\mathrm{MTH1\ WT}}$, 13.23_{G48E}, 1.665_{NUDT15}, 0.7138_{R139C}, $35.00_{\mathrm{PARP1\ WT\text{-}PARP1}}$, $18.07_{\mathrm{L713F\text{-}PARP1}}$, $55.01_{\mathrm{PARP1\ WT\text{-}GFP}}$, $24.03_{\mathrm{L713F\text{-}GFP}}$). In all cases, cells were induced with doxycycline before drug addition.

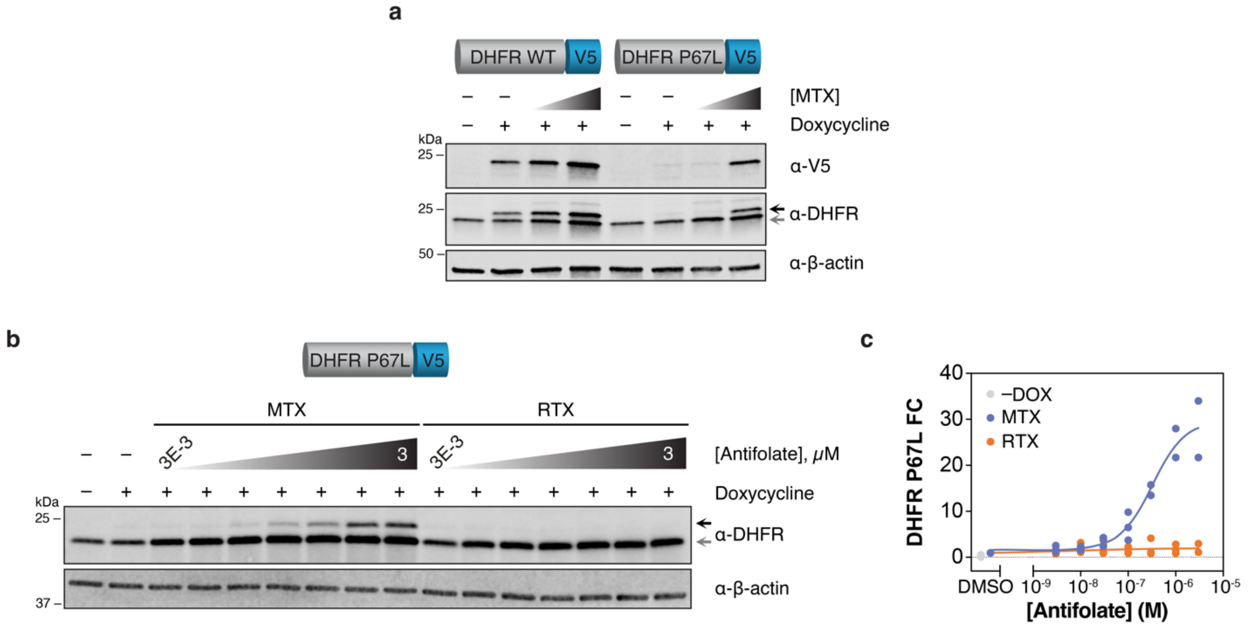

Supplementary Figure 3. DHFR P67L detects intracellular DHFR binders. a, A representative western blot (n=2) comparing WT and P67L DHFR in response to methotrexate. DHFR WT-V5 and DHFR P67L-V5 expression was induced in U-2 OS cells for 24 hours with doxycycline before addition of DMSO, 10 nM, or 1 µM methotrexate (MTX) for 24 hours. Black arrow – exogenous fusion protein; grey arrow – endogenous protein. **b**, A representative western blot (n=3) of DHFR P67L-V5 stabilization by methotrexate (MTX) or raltitrexed (RTX) gradient (3 nM to 3 µM). DHFR P67L-V5 was induced with doxycycline for 24 hours, then incubated with the indicated concentration of anti-folate. Black arrow – exogenous fusion protein; grey arrow – endogenous protein. **c**, Summary quantification of DHFR P67L-V5 abundance following MTX (blue) or RTX (orange) concentration gradient. A no (–) DOX DMSO control (grey) is included for reference. Individual data points for n=3 and respective curve fitting are shown. FC – fold change.

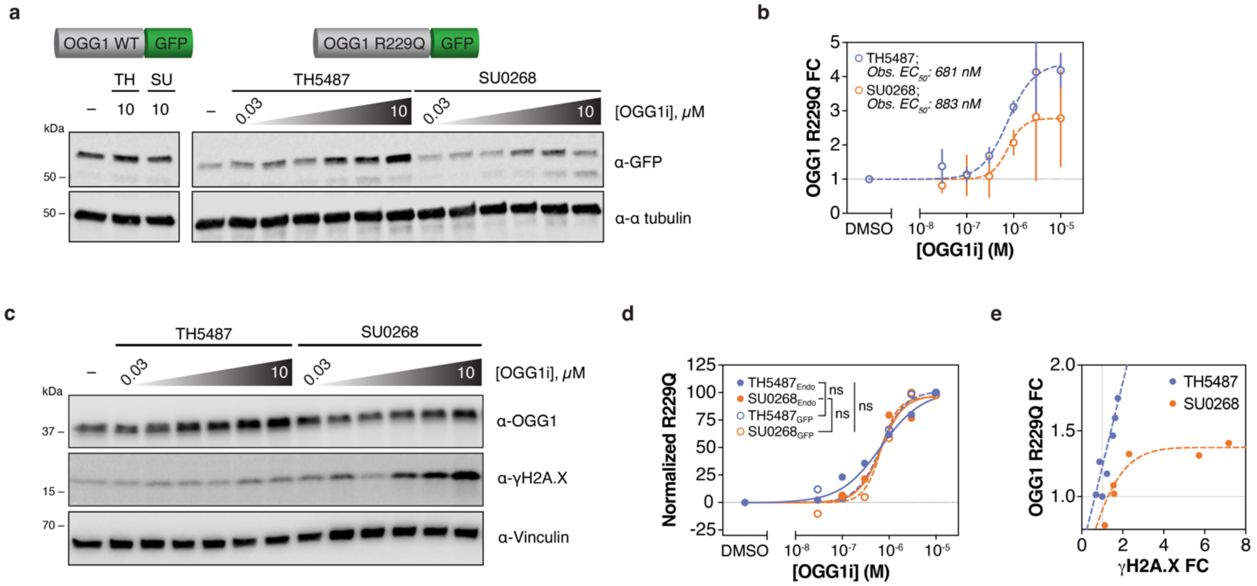

Supplementary Figure 4. Endogenous and exogenous OGG1 R229Q as a biosensor of OGG1 inhibitor binding. a, A representative western blot (n=3) of OGG1 WT-GFP or OGG1 R229Q-GFP responses to TH5487 or SU0268. WT or R229Q-GFP-expressing U-2 OS cells were incubated with DMSO or OGG1i for 24 hours. **b**, Summary quantification of OGG1 R229Q-GFP abundance following TH5487 (blue) or SU0268 (orange) concentration gradient. Means from n=3 ± SD and respective curve fittings are shown. **c**, A representative western blot (n=3) of endogenous OGG1 R229Q stabilization by OGG1i. KG-1 cells were incubated with DMSO or OGG1i for 24 hours. **d**, Overlay of exogenous (open circles) and endogenous (closed circles) OGG1 R229Q following a TH5487 (blue) or SU0268 (orange) concentration gradient normalized to the highest dataset value. Means from n=3 shown with respective curve fittings. ns – not significant by extra sum-of-squares F Test (F: 0.4290, DFn: 3, DFd: 73)$_{all}$, (F: 0.5406, DFn: 1, DFd: 33)$_{SU0268}$, (F: 0.1861, DFn: 1, DFd: 32)$_{TH5487}$. P_{all}=0.6197, P_{SU0268}=0.4552, P_{TH5487}=0.9021. **e**, Two-dimensional summary of R229Q stabilization and γH2A.X induction following TH5487 (blue) or SU0268 (orange) concentration gradients. Means of n=3 shown along with linear (TH5487, $y = 0.5863x + 0.5846$) or non-linear (logistic growth [r^2:0.4464]; SU0268) lines-of-best-fit. FC – fold change.

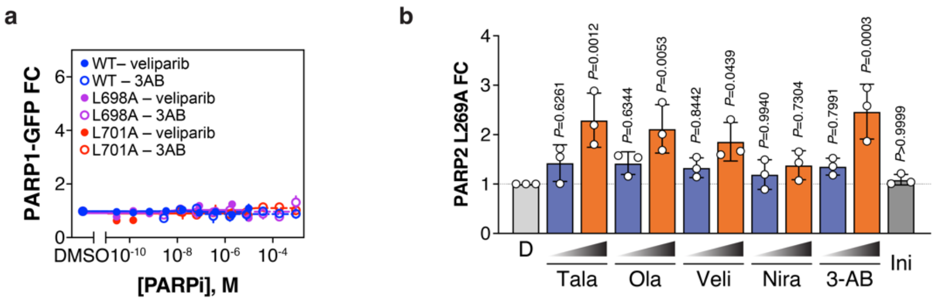

Supplementary Figure 5. Non-permissive PARP1 CeTEAM variants and rational expansion to PARP2 L269A. a, Live-cell fluorescence fold change of WT (blue), L698A (purple), or L701A (red) PARP1-GFP CeTEAM variants with veliparib (closed circles) or 3-AB (open circles) dose-response after 24 hours. Means of n=2 experiments shown ± range. **b**, Summary western blot quantitation data of PARP2 L269A-GFP fold change following low (blue) or high (orange) concentration incubation of talazoparib, olaparib, veliparib, niraparib (10 nM [blue]/1 µM [orange]), or 3-aminobenzamide (3-AB, 10 µM [blue]/1 mM [orange]) incubation for 24 hours and relative to DMSO control (light grey). 20 µM iniparib is included as a negative control (dark grey). Mean of n=3 ± SD. *P* values shown for comparisons by ordinary one-way ANOVA with multiple comparisons to the DMSO control (Dunnett's test; $F_{Treatment}$ [DFn, DFd] = 5.842 [11, 24]). RFU – relative fluorescence units, FC – fold change.

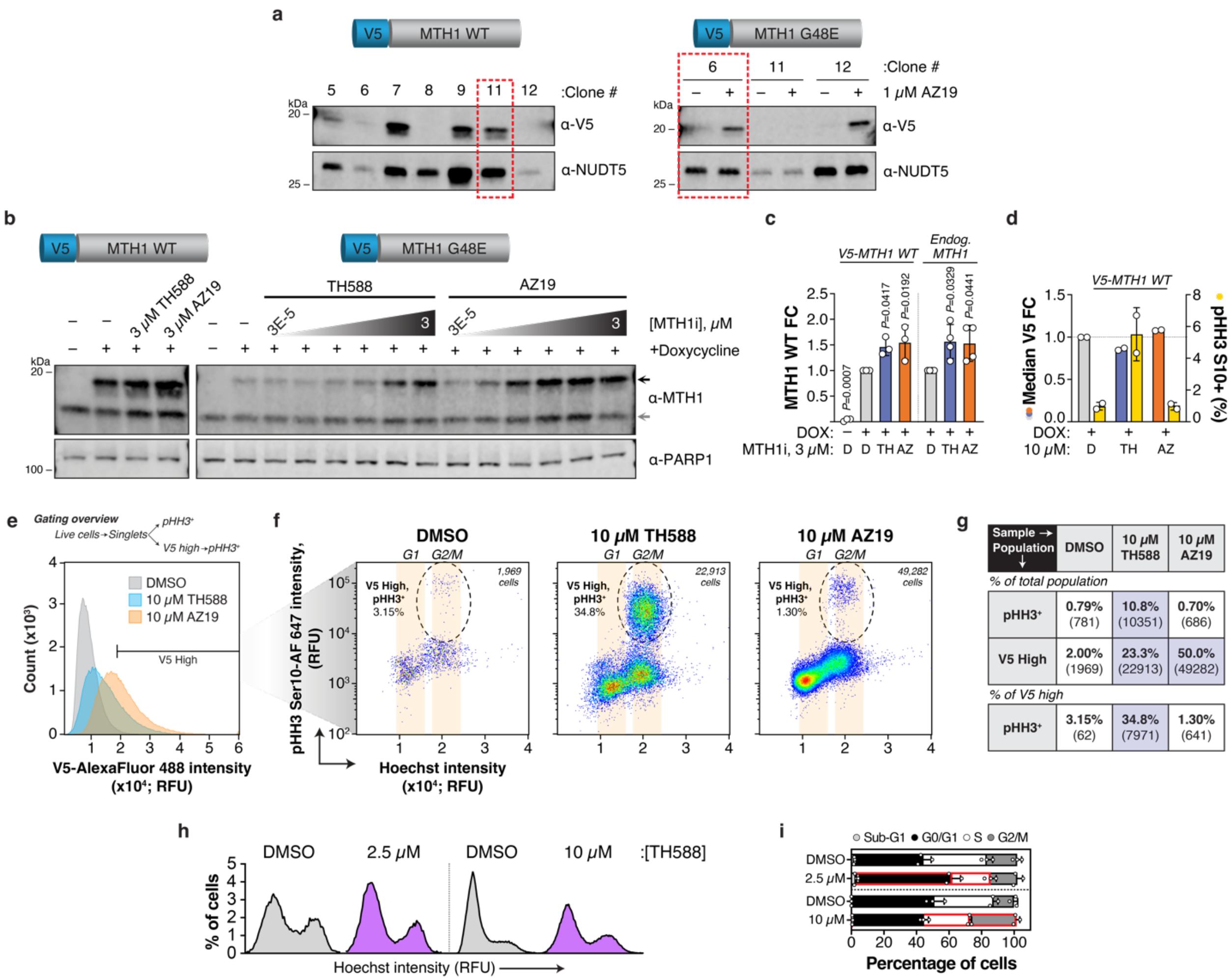

Supplementary Figure 6. Optimization and characterization of a MTH1 G48E drug biosensor. **a**, Clonal selection of WT (clone 11) and G48E (clone 6) MTH1 cells. **b**, A representative blot (n=3) of MTH1 G48E saturation curves generated with either TH588 or AZ19 after 24-hour incubation following doxycycline induction. WT MTH1 included for comparison. Black arrow – exogenous fusion protein; grey arrow – endogenous protein. **c**, Summary western blot quantitation of exogenous (left) or endogenous (right) MTH1 WT after DMSO (light grey), 3 µM TH588 (blue), or 3 µM AZ19 (orange) for 24 hours. Means of n=3 (exogenous) or n=4 (endogenous) ± SD. *P* values shown for one-way ANOVA with multiple comparisons to the DMSO control (Dunnett's test; $F_{\text{Treatment (c left)}}$ [DFn, DFd] = 39.86 [3, 8], $F_{\text{Treatment (c right)}}$ [DFn, DFd] = 5.185 [2, 9]. **d**, Flow cytometry quantitation of V5-MTH1 WT (left axis) and pHH3 Ser10^{+} (%, right axis, yellow) following 24-hour, 10 µM MTH1i. Means of n=2 ± range. **e**, Representative histograms of V5-G48E intensity and V5 high events following 24-hour, 10 µM TH588 (blue) or AZ19 (orange). V5 high events arbitrarily defined as intensity >top 2% of DMSO control. Concise gating overview shown (detailed version in **Supplementary Figure 18a**). **f**, V5 high cells plotted by pHH3 Ser10 (y-axis) and Hoechst intensity (x-axis). V5 high/pHH3^{+} double positive populations indicated. **g**, Proportion of pHH3^{+} and V5 high cells from total singlet population, and of pHH3^{+} cells in the V5 high population (%). Events in parentheses and notable differences highlighted in blue. **e**/**f**/**g** are representative of n=3. **h**, Representative (from n=3) cell cycle

histograms comparing 2.5 or 10 μM TH588 (purple) to respective DMSO (grey) controls after 24 hours. $DMSO_{2.5μM}$=19,706 cells; $TH588_{2.5μM}$=19,429 cells; $DMSO_{10μM}$=99,817 cells; and $TH588_{10μM}$=99,623 cells. **i**, Cell cycle proportions from **h**. Mean of n=3 ± SD; 10 μM dataset is also presented in **Figure 3f**. Red highlights – $P < 0.05$ by multiple unpaired t test with Welch's correction ($P_{2.5,Sub\text{-}G1}$=0.57070, $P_{2.5,\ G0/G1}$=0.039167, $P_{2.5,S}$=0.021621, $P_{2.5,G2/M}$=0.5707, $P_{10,Sub\text{-}G1}$=0.04455, $P_{10,G0/G1}$=0.12546, $P_{10,S}$=0.04455, $P_{10,G2/M}$=0.002302). *2.5 μM*; t ratio/df = $1.193/2.215_{Sub\text{-}G1}$, $4.257/3.980_{G1/G0}$, $5.466/4.000_{S}$, $0.9586/3.914_{G2/M}$; *10 μM*; t ratio/df = $7.069/2.003_{Sub\text{-}G1}$, $2.119/2.961_{G1/G0}$, $5.324/2.823_{S}$, $9.956/3.994_{G2/M}$. FC – fold change.

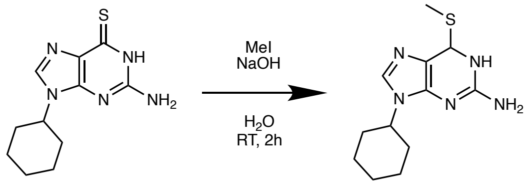

Supplementary Figure 7. Synthesis of TH008228 (9-cyclohexyl-6-(methylthio)-9H-purin-2-amine). Refer to Methods section for details.

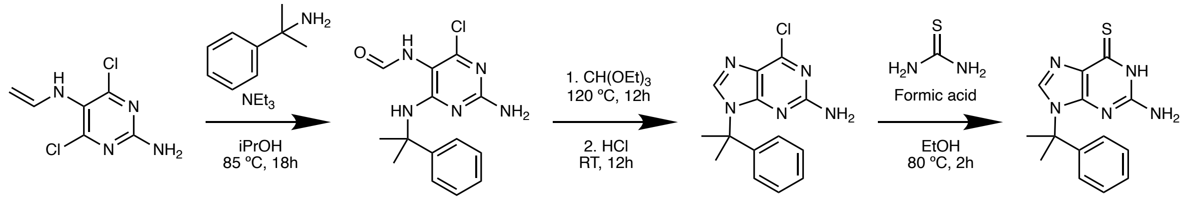

Supplementary Figure 8. Synthesis of TH008234 (2-amino-9-(2-phenylpropan-2-yl)-3H-purine-6(9H)-thione). Refer to Methods section for details.

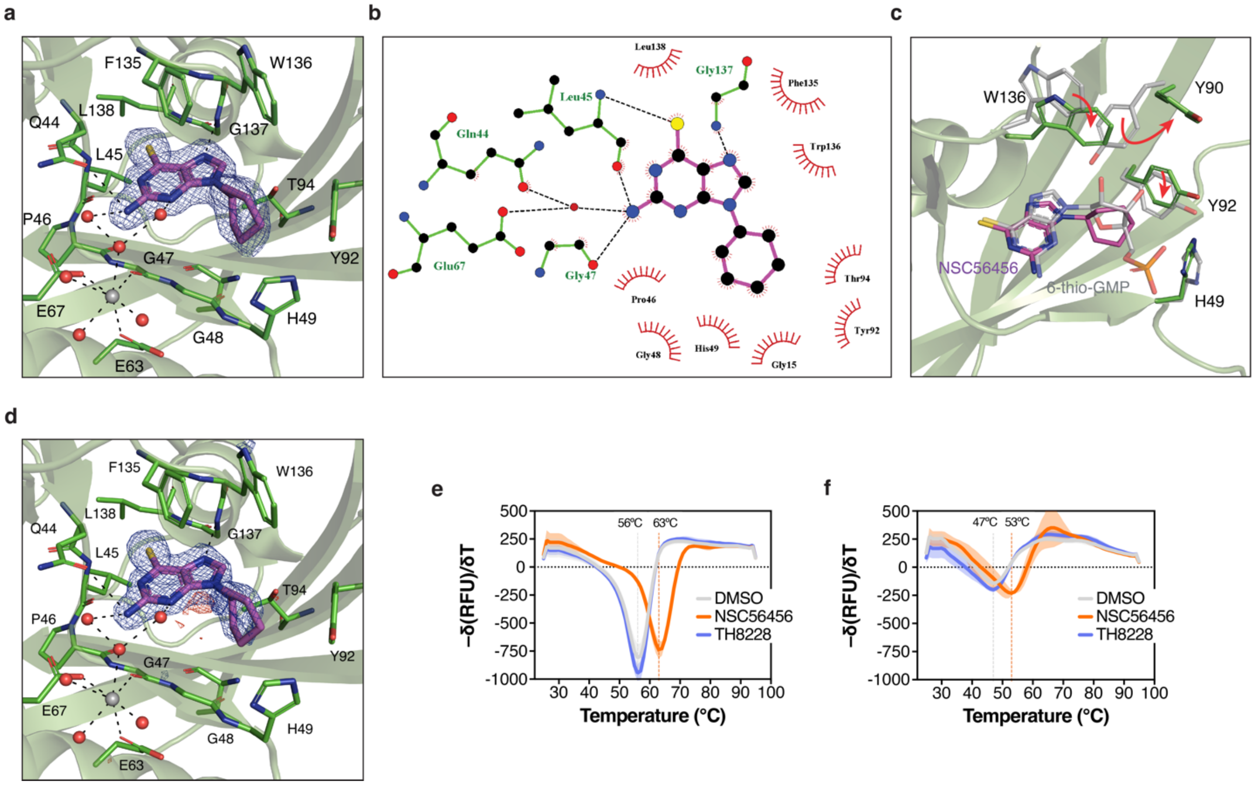

Supplementary Figure 9. NSC56456, but not the inactive analog, TH8228, binds to NUDT15 WT and the R139C missense variant. a, Co-crystal structure of NSC56456 bound to the catalytic site of WT NUDT15 with binding interactions. NSC56456 is depicted in magenta with $2F_o$-F_c electron density map in blue contoured at 1.0 σ), interacting NUDT15 residues are in bright green, magnesium ions in grey, and water molecules in red. Hydrogen bonding interactions are depicted by dashed, black lines. **b**, Ligplot+ representation of NSC56456 binding interactions in the NUDT15 active site. Residues interacting with NSC56456 (magenta) are shown in green. Hydrophobic interactions are shown as an arc with spokes and hydrogen bonds as dashed lines. **c**, Binding modality comparison of NSC56456 and 6-thio-GMP within NUDT15. The aligned 6-thio-GMP-bound structure of NUDT15 (PDB ID: 5LPG) is shown in grey, while the NUDT15-NSC56456 structure is green. Residues that undergo a significant conformational change between the two structures are highlighted. The 6-thioguanine group is positioned in nearly the exact same position in both structures, aside from a slight tilt. This tilt strengthens several hydrogen bonds by bringing the bonding partners into closer proximity (bonding to Leu45 is decreased by 0.1 Å and Gly137 by 0.4 Å, respectively, **b**). The cyclohexane ring is situated similarly to ribose in the 6-thio-GMP structure, but since it lacks the protruding hydroxyl groups, this leaves empty space in the binding pocket. Trp136 has moved into the active site by 2.9 Å to fill this empty space, which causes Tyr90 to be flipped outwards. **d**, F_o-F_c omit map contoured at 4.0 σ (blue) and -4.0 σ (red). **e**, DSF of NUDT15 WT with 50 µM NSC56456 (orange) or TH8228 (blue) compared to DMSO (grey) and displayed as the negative first derivative. Means of n=2 independent experiments ± range (shading) shown. **f**, DSF of NUDT15 R139C with 50 µM NSC56456 (orange) or TH8228 (blue) compared to DMSO (grey) and displayed as the negative first derivative. Means of n=2 ± range (shading) shown. **e** and **f** are related to **Figure 4e**.

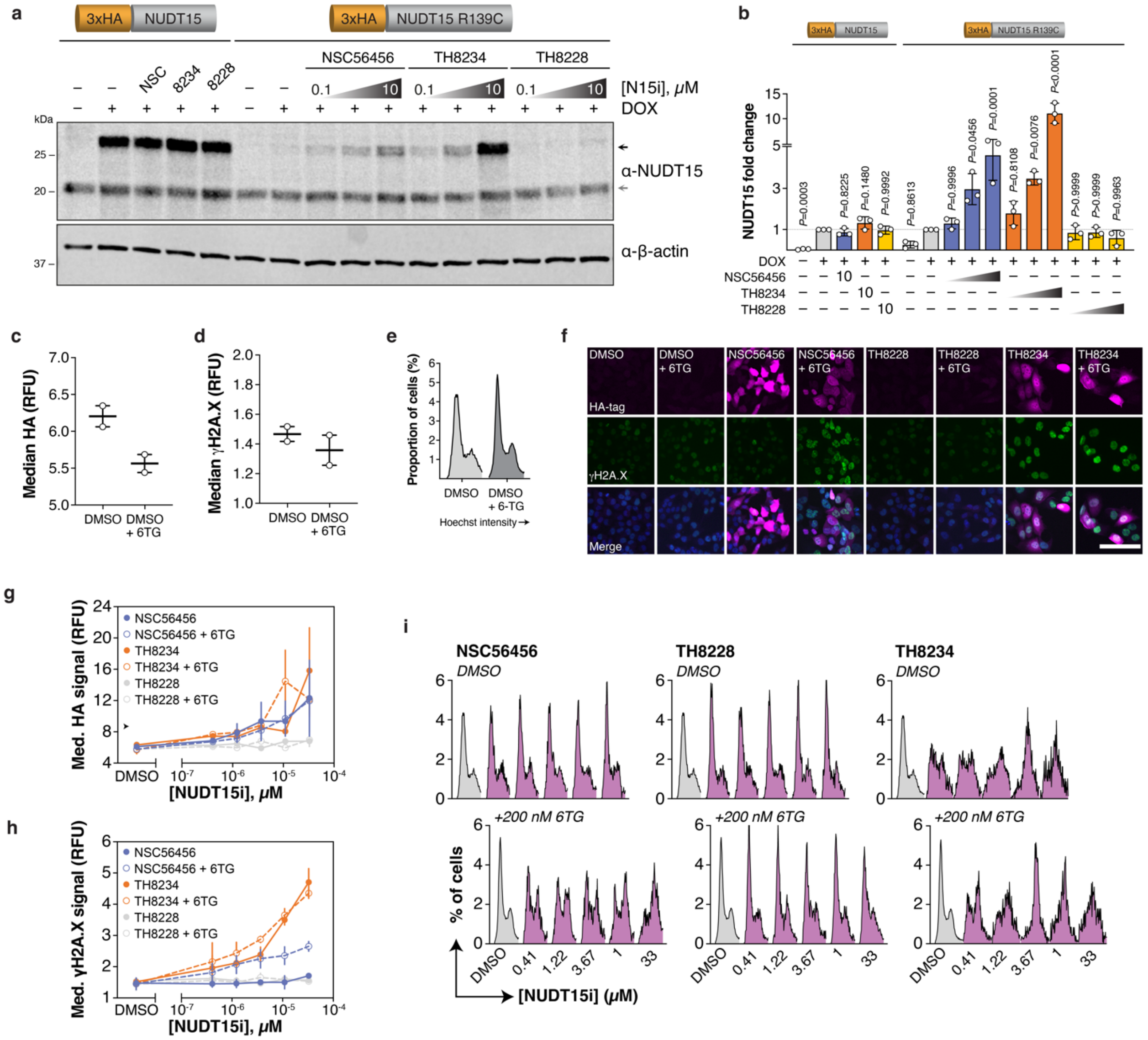

Supplementary Figure 10. Establishing NUDT15 R139C as a thiopurine biosensor. a, A representative western blot (n=3) of NUDT15 WT and R139C accumulation after 0.1, 1, or 10 μM NUDT15i (24 hours). 10 μM NUDT15is were used for WT cells. Black arrow – exogenous fusion protein; grey arrow – endogenous protein. **b**, 3xHA-NUDT15 WT or R139C abundance following NSC56456 (blue), TH8234 (orange), or TH8228 (yellow) concentration gradient and compared to DMSO (light grey). Means from n=3 ± SD are shown. *P* values shown for comparisons by one-way ANOVA with multiple comparisons to the WT DMSO or R139C DMSO control (Dunnett's test; $F_{\text{Treatment WT}}$ [DFn, DFd] = 20.69 [4, 10], $F_{\text{Treatment R139C}}$ [DFn, DFd] = 45.64 [10, 22]). **c**, Median HA and (**d**) γH2A.X intensities of individual 3xHA-NUDT15 R139C cells after DMSO or 200 nM 6TG (72 hours). Mean of n=2 ± range shown. **e**, Representative Hoechst histograms related to **c** and **d**. Cell cycle profiles inferred from n_{DMSO}: 25,371 or n_{6TG}: 47,435 cells. **f**, Representative micrographs (from n=2) of induced HA-R139C cells pre-treated with DMSO, 33 μM NSC56456, 33 μM TH8228, or 33 μM TH8234, then -/+ 6TG for 72 hours, and stained with indicated markers. Hoechst staining shown in the merged image. Scale bar=100 μm. **g**, Median HA or γH2A.X (**h**) intensity of induced HA-R139C cells pre-incubated with NSC56456 (blue), TH8234 (orange), or

TH8228 (grey) for 3 hours then DMSO (closed circles) or 200 nM 6TG (open circles) for another 72 hours. Means of n=2 and data range shown (1000 cells per sample). **i**, Representative cell cycle profiles of samples treated as above (n=2). See Source Data for cell numbers analyzed. RFU – relative fluorescence units, purple histogram – treated with NUDT15i.

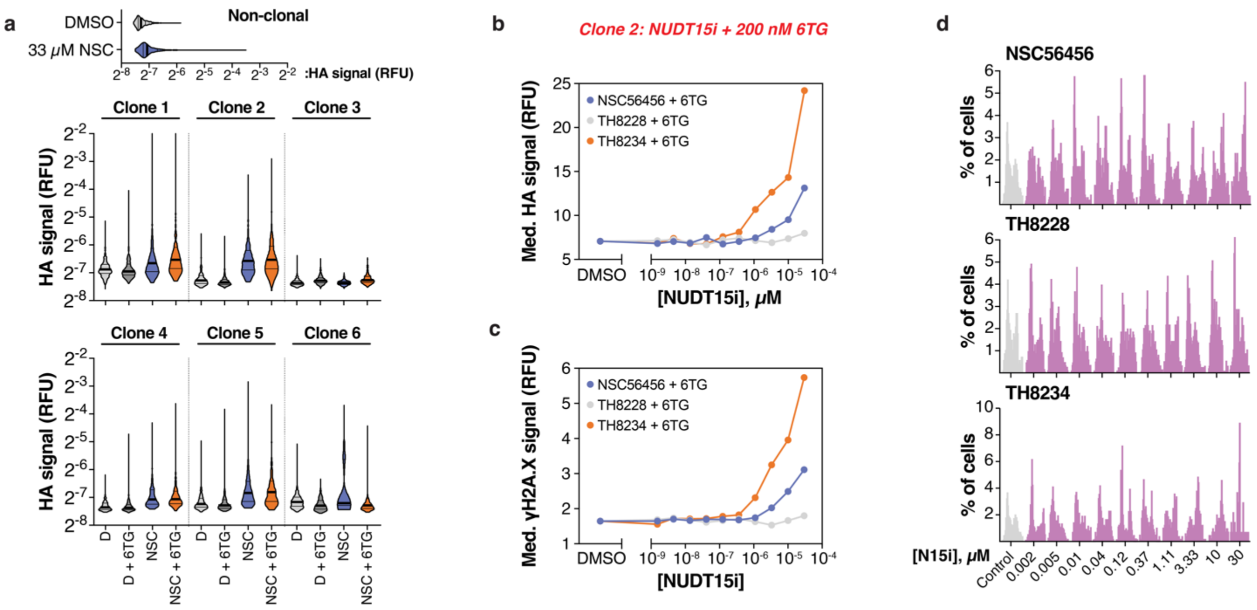

Supplementary Figure 11. Optimized stabilization dynamic range with 3xHA-NUDT15 R139C clones. a, HA signal intensity violin plots of induced 3xHA-NUDT15 R139C clonal cells after DMSO or 30 μM NSC56456 -/+ 200 nM 6TG (72 hours). n=500 cells per sample. Inset – non-clonal cells for comparison. Medians (thick lines) and quartiles (thin lines) are shown. **b**, Representative data of median HA and γH2A.X (**c**) signal from 500 representative cells following pre-incubation of induced clone 2 cells with NSC56456 (blue), TH8234 (orange), or TH8228 (grey) followed by 200 nM 6TG. **d**, Cell cycle profiles (Hoechst histograms) for clone 2 cells (related to **b** and **c**). See Source Data for cell numbers analyzed. RFU – relative fluorescence units, purple histogram – treated with NUDT15i.

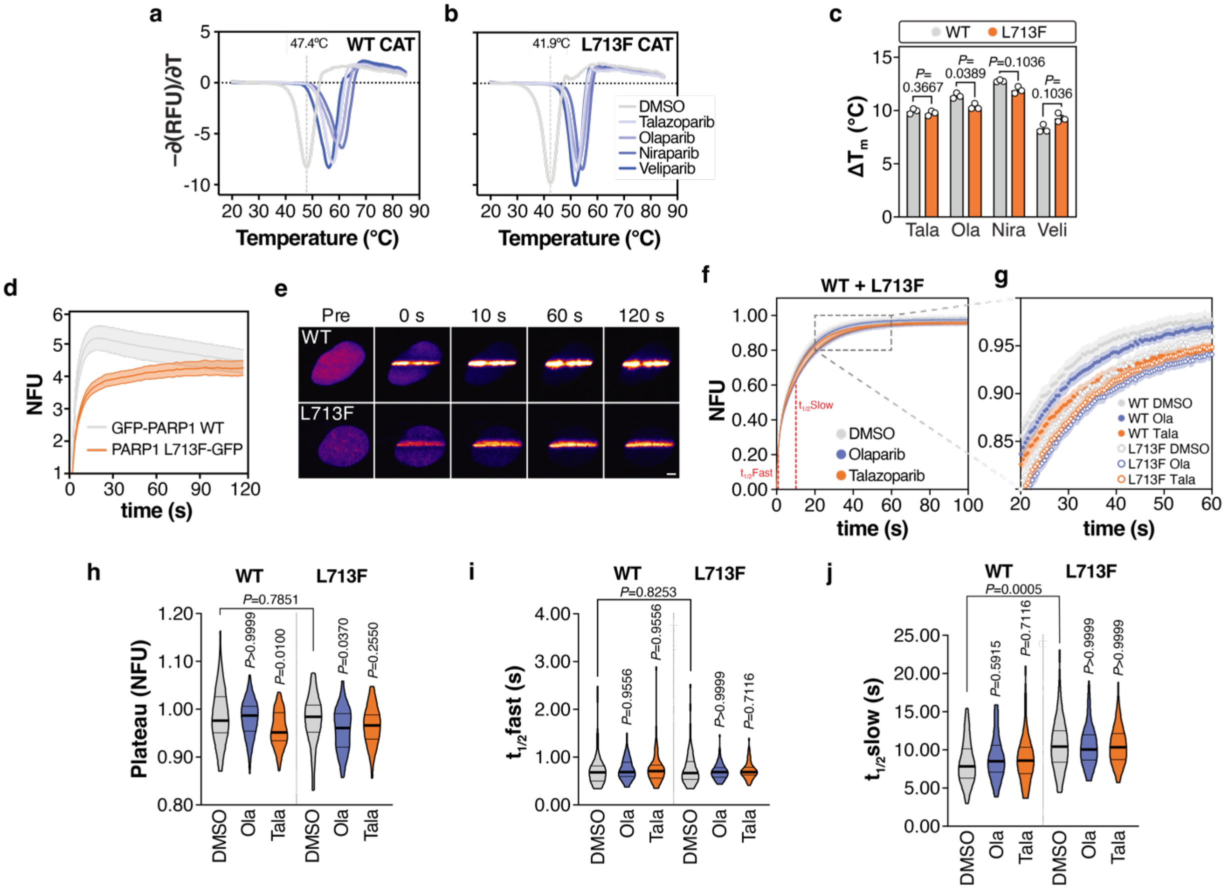

Supplementary Figure 12. Biophysical characterization of PARPi binding to, DNA damage recruitment of, and mobility of PARP1 L713F. a, Representative DSF with 5 µM PARP1 WT or (**b**) L713F catalytic domain with DMSO or 250 µM PARPi displayed as the negative first derivative. Melting temperatures (T_m) of DMSO controls are indicated. **c**, T_m change with PARPi relative to DMSO (ΔT_m). Means of n=3 ± SD shown. **d**, Quantified live-cell recruitment kinetics for GFP-PARP1 WT and PARP1 L713F-GFP to microirradiation-induced DNA damage sites. Means ± SEM are shown from 20 (WT) or 19 total cells (L713F; two independent experiments). **e**, Representative PARP1 saturation micrographs following microirradiation (from **d)**. Scale bar = 5 µm. **f**, FRAP profiles of PARP1 WT and L713F-GFP with PARPi. GFP-PARP1 WT or L713F-GFP-expressing U-2 OS cells after 24-hour DMSO, 5 µM olaparib, or 0.5 µM talazoparib prior to FRAP. Approximate half-time for fast and slow components of FRAP curve shown. Total of 45 (WT, DMSO), 49 (L713F, talazoparib) or 50 cells (all others) from 5 independent experiments. **g**, Zoomed inset from **f**. **h**, Plateau, fast half-time ($t_{1/2}$fast; **i**), and slow half-time ($t_{1/2}$slow; **j**) from FRAP experiments of WT or L713F cells treated with DMSO (grey), olaparib (blue), or talazoparib (orange). *P* values shown for comparisons by multiple unpaired t test with Welch's correction (Holm-Šídák multiple comparison test; **b;** t ratio/df = $1.017/3.991_{\text{tala}}$, $4.641/3.977_{\text{ola}}$, $4.444/2.297_{\text{nira}}$, $3.131/3.946_{\text{veli}}$), Kruskal-Wallis analysis with comparisons to DMSO treatments for each group (Dunn's test; **h**, **i**, and **j**; Kruskal-Wallis statistic = $10.87_{\mathbf{h},\text{WT}}$, $5.713_{\mathbf{h},\text{L713F}}$, $0.8287_{\mathbf{i},\text{WT}}$, $0.8899_{\mathbf{i},\text{L713F}}$, $1.218_{\mathbf{j},\text{WT}}$, $0.08776_{\mathbf{j},\text{L713F}}$), or two-tailed Mann-Whitney U test (**h**, **i**, and **j** [comparison of WT and L713F DMSO controls]; Mann-Whitney U = $1088_{\mathbf{h}}$, $1095_{\mathbf{i}}$, $664_{\mathbf{j}}$). NFU – normalized fluorescence units, RFU – relative fluorescence units.

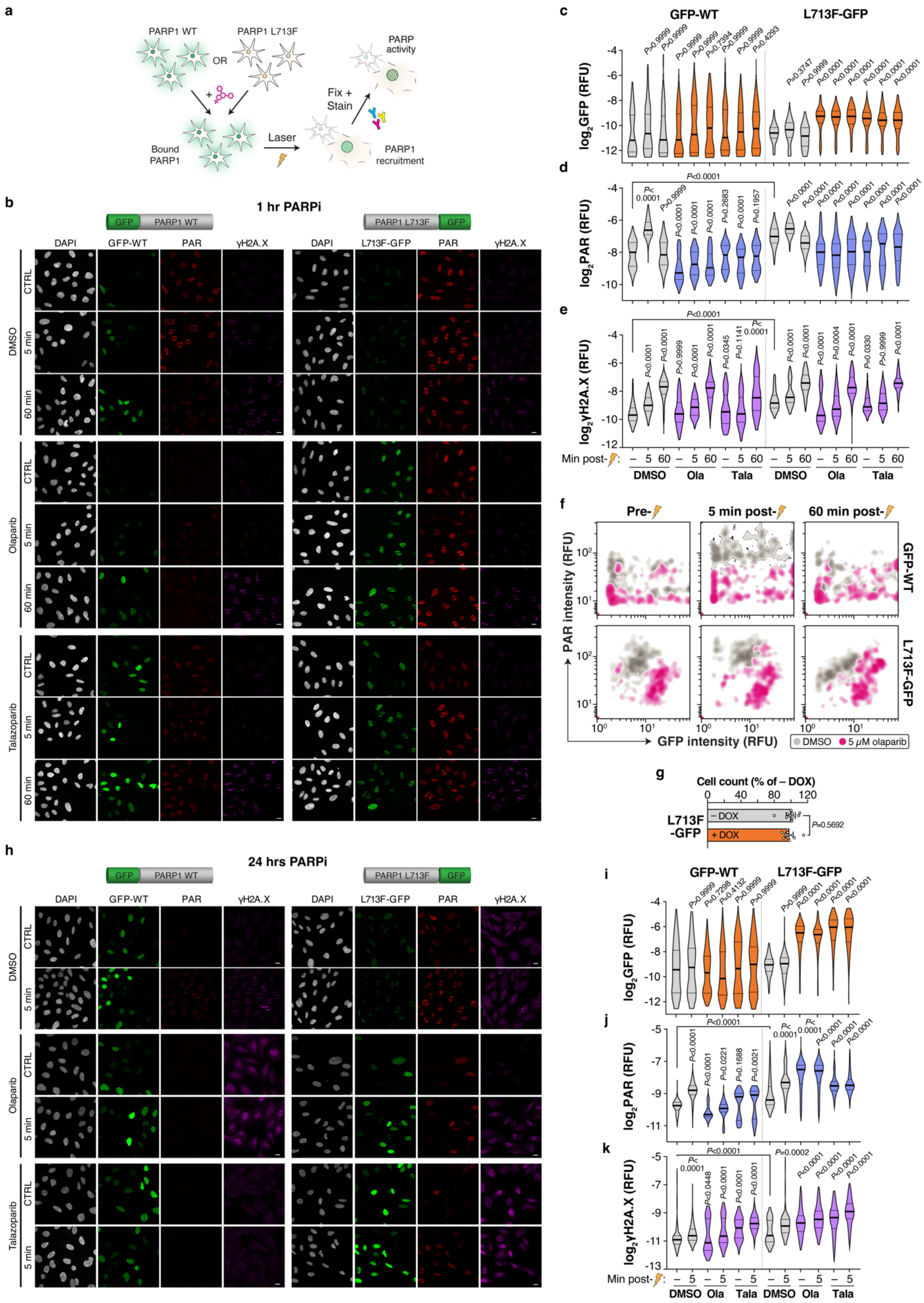

a
PARP1 WT
PARP1 L713F
OR
Bound PARP1
Laser
Fix + Stain
PARP activity
PARP1 recruitment
b
1 hr PARPi
24 hrs PARPi
DAPI
GFP-WT
L713F-GFP
PAR
γH2A.X
DMSO
Olaparib
Talazoparib
CTRL
5 min
60 min
c
GFP-WT
L713F-GFP
log2GFP (RFU)
d
log2PAR (RFU)
e
log2γH2A.X (RFU)
Min post-
DMSO
Ola
Tala
f
Pre-
5 min post-
60 min post-
PAR intensity (RFU)
GFP intensity (RFU)
DMSO
5 μM olaparib
g
Cell count (% of – DOX)
L713F -GFP
– DOX
+ DOX
P=0.5692
h
i
j
k

Supplementary Figure 13. PARP1 L713F adheres to PARP1 biology in DNA damage repair and response to PARPi. a, Monitoring PARP-GFP intensity and activity at DNA damage sites with PARPi. U-2 OS GFP-PARP1 WT or PARP1 L713F-GFP cells were induced with DOX and pre-treated with DMSO, 5 µM olaparib, or 0.5 µM talazoparib for 1 or 24 hours before microirradiation and fixation after 5 or 60 minutes. **b**, Representative micrographs of GFP-PARP1 WT, L713F-GFP, PAR, and γH2A.X recruitment dynamics to sites of microirradiation after 1 hour of PARPi. For all micrographs, DAPI – blue; GFP-PARP1 – green; PAR – red; γH2A.X – magenta and scale bar = 20 µm. **c**, Violin plots of log_2-transformed GFP, PAR (**d**), and γH2A.X (**e**) intensity following 1-hour PARPi incubation and post-microirradiation (5 and 60 minutes). The medians (thick lines) and quartiles (thin lines) are shown for each plot, while DMSO-treated samples are represented in grey and PARPi-treated counterparts are colored. 229 total cells per group (three independent experiments). f, Two-dimensional dynamics of WT or L713F PARP1-GFP stabilization and PAR abundance in the absence (grey) or presence (magenta) of olaparib after 1 hour (related to **c**/**d**). **g**, Normalized counts from PARP1 L713F-GFP #5 cells following doxycycline (48 hours). Means of 11 total replicates (two independent experiments) with 95% confidence intervals shown. **h**, Representative micrographs after 24 hours of PARPi, as above. **i**, Violin plots of log_2-transformed GFP, PAR (**j**), and γH2A.X (**k**) intensity following 24-hour PARPi and microirradiation (5 minutes). Plots are identical as above. 282 total cells (except L713F-Tala-5min: 208 cells) from three independent experiments. *P* values shown for comparisons by Kruskal-Wallis analysis with multiple comparisons to pre-irradiated DMSO treatments for each group (Dunn's test; **c**-**e** and **i**-**k**; Kruskal-Wallis statistic: $12.33_{\mathbf{c},WT}$, $533.8_{\mathbf{c},L713F}$, $726.7_{\mathbf{d},WT}$, $572.4_{\mathbf{d},L713F}$, $652.9_{\mathbf{e},WT}$, $836.2_{\mathbf{e},L713F}$, $12.25_{\mathbf{i},WT}$, $821.7_{\mathbf{i},L713F}$, $498.9_{\mathbf{j},WT}$, $352.5_{\mathbf{j},L713F}$, $300.1_{\mathbf{k},WT}$, $293.6_{\mathbf{k},L713F}$), two-tailed Mann-Whitney U test (**d**, **e**, **j**, and **k** [comparison of WT and L713F pre-irradiated DMSO controls], Mann-Whitney U = $7787_{\mathbf{d}}$, $8464_{\mathbf{e}}$, $19116_{\mathbf{j}}$, $26745_{\mathbf{k}}$), or two-tailed t test (**g**; t, df = 0.5787, 20). RFU – relative fluorescence units.

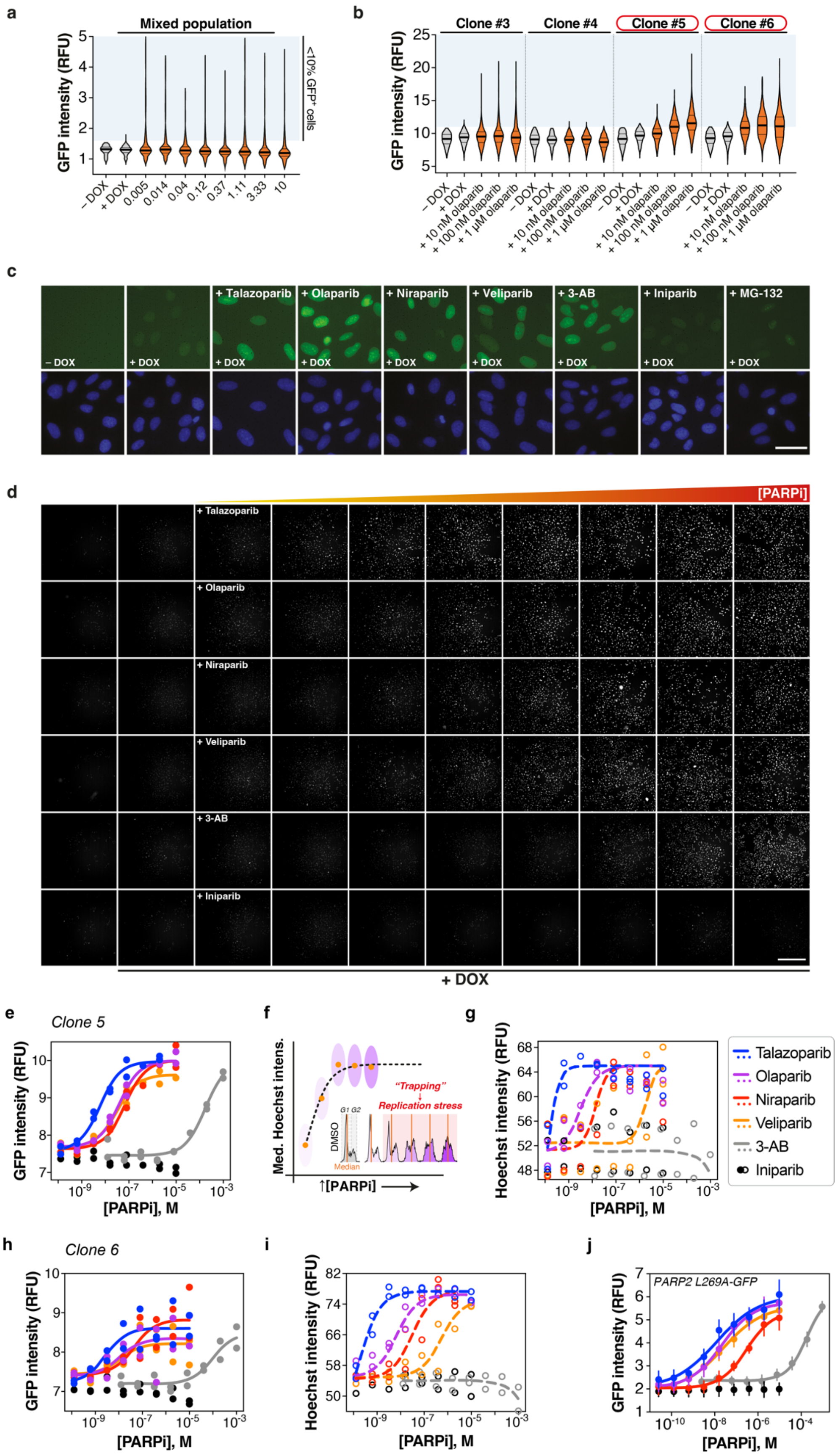
a
Mixed population
GFP intensity (RFU)
<10% GFP+ cells
– DOX
+ DOX
0.005
0.014
0.04
0.12
0.37
1.11
3.33
10
b
Clone #3
Clone #4
Clone #5
Clone #6
GFP intensity (RFU)
– DOX
+ DOX
+ 10 nM olaparib
+ 100 nM olaparib
+ 1 μM olaparib
c
+ Talazoparib
+ Olaparib
+ Niraparib
+ Veliparib
+ 3-AB
+ Iniparib
+ MG-132
d
[PARPi]
+ DOX
e
Clone 5
GFP intensity (RFU)
[PARPi], M
f
Med. Hoechst intens.
"Trapping"
Replication stress
DMSO
Median
G1 G2
↑[PARPi]
g
Hoechst intensity (RFU)
[PARPi], M
Talazoparib
Olaparib
Niraparib
Veliparib
3-AB
Iniparib
h
Clone 6
GFP intensity (RFU)
[PARPi], M
i
Hoechst intensity (RFU)
[PARPi], M
j
PARP2 L269A-GFP
GFP intensity (RFU)
[PARPi], M

Supplementary Figure 14. Clonal characterization of live PARP1 L713F-GFP cells. a, Representative violin plots of live-cell GFP intensity from heterogeneous U-2 OS PARP1 L713F-GFP cells after 24 hours of a talazoparib gradient. 501 cells per condition. **b**, Representative violin plots of live-cell GFP intensity from clonal PARP1 L713F-GFP cells after 24 hours of an olaparib gradient. Clone 5 and 6 were chosen for follow-up study (red). 499 cells per condition. For violin plots in **a** and **b**, medians (thick lines) and quartiles (thin lines) are shown, while DMSO-treated samples are represented in grey and PARPi-treated counterparts are orange. Blue regions are a visual reference for population shifts. **c**, Representative live-cell GFP and Hoechst micrographs of clone 5 cells induced with doxycycline and incubated with 10 µM talazoparib, olaparib, niraparib, veliparib, 1000 µM 3-aminobenzamide (3-AB), or 10 µM iniparib for 24 hours. 5 µM MG-132 for 6 hours was used as a control. Scale bar = 50 µm. **d**, Representative live-cell GFP micrographs (greyscale) from a dose-response of talazoparib, olaparib, niraparib, veliparib, 3-AB, and iniparib of induced clone 5 cells after 24 hours. Scale bar = 500 µm. **e**, Summary median GFP intensity saturation curves from clone 5 cells. Means from n=2 and respective curve fittings are shown. **f**, Graphic describing the use of median Hoechst intensity (DNA content) as a readout of PARPi-induced, trapping-related replication stress. **g**, Hoechst intensity (DNA content) saturation curves from clone 5 cells. Means from n=2 and respective curve fittings are shown. **h**, Median GFP intensity and Hoechst intensity (DNA content, **i**) saturation curves from clone 6 cells. Means from n=2 and respective curve fittings are shown. **j**, Median GFP intensity saturation curves with U-2 OS cells constitutively expressing PARP2 L269A-GFP (extended dataset related to **Figure 2i**). Means from n=5 ± SEM and respective curve fittings are shown. RFU – relative fluorescence units.

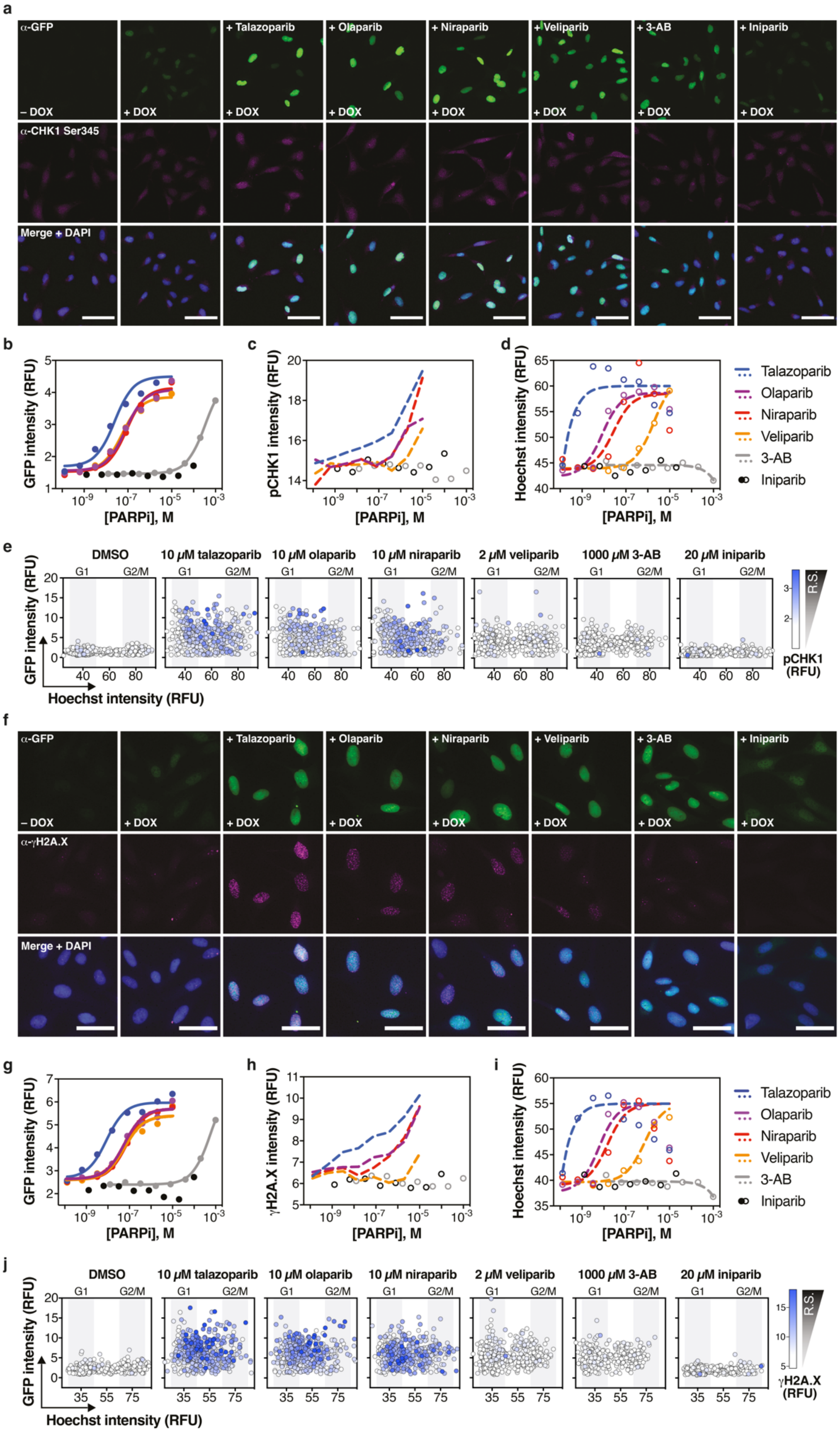

a
α-GFP
α-CHK1 Ser345
Merge + DAPI
– DOX
+ DOX
+ Talazoparib
+ Olaparib
+ Niraparib
+ Veliparib
+ 3-AB
+ Iniparib
b
GFP intensity (RFU)
[PARPi], M
c
pCHK1 intensity (RFU)
d
Hoechst intensity (RFU)
Talazoparib
Olaparib
Niraparib
Veliparib
3-AB
Iniparib
e
DMSO
10 μM talazoparib
10 μM olaparib
10 μM niraparib
2 μM veliparib
1000 μM 3-AB
20 μM iniparib
G1
G2/M
R.S.
pCHK1 (RFU)
Hoechst intensity (RFU)
f
α-γH2A.X
g
h
γH2A.X intensity (RFU)
i
j
γH2A.X (RFU)

Supplementary Figure 15. Multiparametric interrogation of replication stress in fixed PARP1 L713F-GFP cells exposed to PARP inhibitors. **a**, Representative micrographs of induced U-2 OS PARP1 L713F-GFP clone 5 cells incubated with increasing concentrations of talazoparib, olaparib, niraparib, veliparib, iniparib, or 3-AB for 24 hours and stained for GFP (target engagement), CHK1 Ser345 (pCHK1; replication stress/DNA damage), and Hoechst 33342 (DNA content; in merged image). Scale bar – 200 μm. **b**, Median GFP, CHK1 Ser345 (**c**), and Hoechst (**d**) intensities. **e**, Per-cell, three-dimensional visualization of GFP (y-axis), Hoechst (x-axis), and CHK1 Ser345 (blue gradient) after DMSO, 10 μM talazoparib, 10 μM olaparib, 10 μM niraparib, 2 μM veliparib, 1000 μM 3-AB or 20 μM iniparib. Estimated G1 and G2/M cell populations are demarcated. n=500 cells shown per condition. **f**, Representative micrographs of induced clone 5 cells incubated with increasing concentrations of talazoparib, olaparib, niraparib, veliparib, iniparib, or 3-AB for 24 hours and stained for GFP, γH2A.X (replication stress/DNA damage), and Hoechst 33342 (in merged image). Scale bar – 50 μm. **g**, Median GFP, γH2A.X (**h**), and Hoechst (**i**) intensities. **j**, Per-cell three-dimensional visualization of GFP (y-axis), Hoechst (x-axis), and γH2A.X (blue gradient) after DMSO, 10 μM talazoparib, 10 μM olaparib, 10 μM niraparib, 2 μM veliparib, 1000 μM 3-AB or 20 μM iniparib. Estimated G1 and G2/M cell populations are demarcated. 500 cells per condition are shown. In all cases, representative experiments (from n=2) and lines of best fit are shown; RFU – relative fluorescence units and blue gradient – increasing replication stress/DNA damage (CHK1 Ser345 or γH2A.X; R.S.).

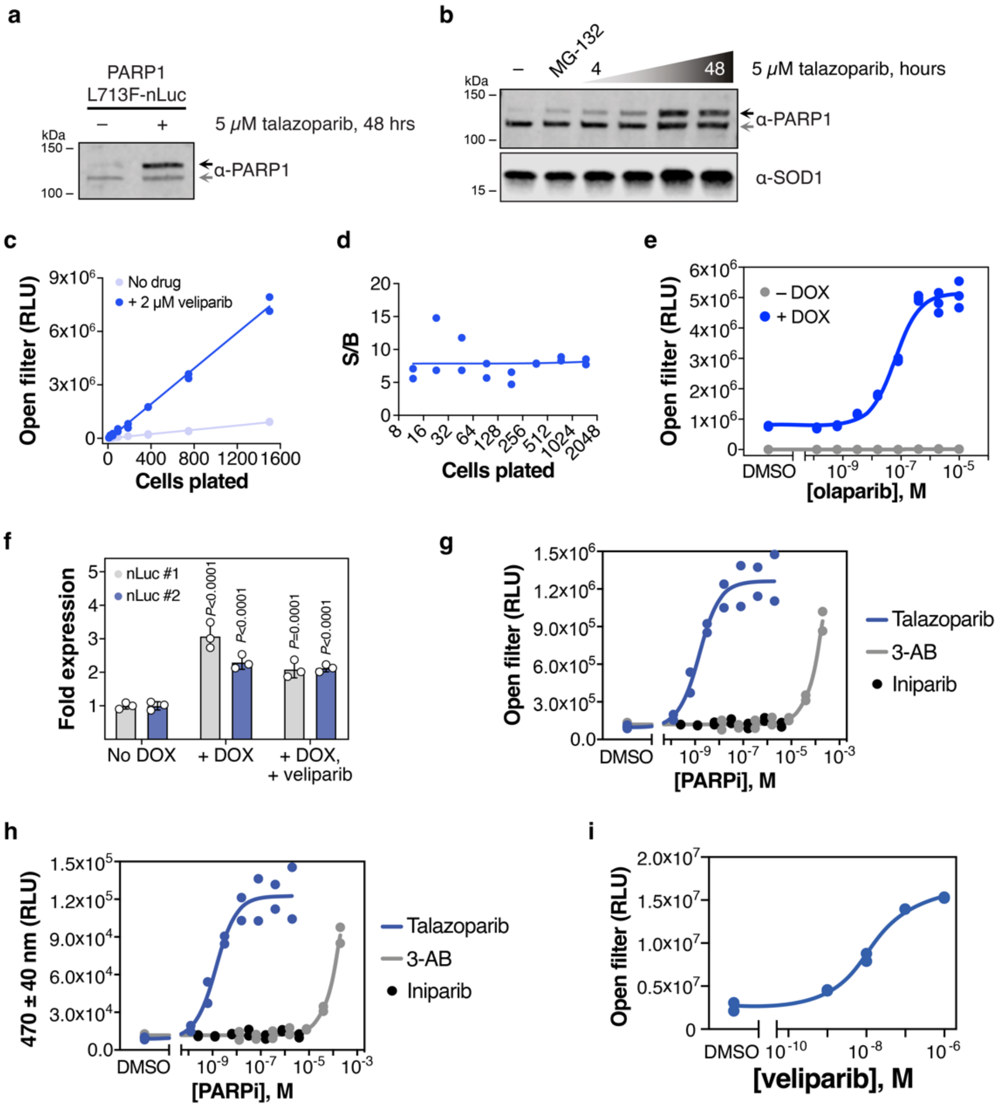

Supplementary Figure 16. Assessment of PARP1 L713F-nLuc stabilization by PARPi. a, U-2 OS PARP1 L713F-nLuc #3 cells pre-treated with doxycycline were exposed to DMSO or 5 µM talazoparib for 48 hours. **b**, A representative blot (n=2) of cells treated with 5 µM talazoparib for 4-48 hours (as in **a**). DMSO and 5 µM MG-132 controls were added for 48 and 6 hours, respectively. Black arrows – PARP1 L713F-nLuc; grey arrows – endogenous PARP1. **c**, Cells pre-treated as in **a**, plated at different densities, and treated with DMSO (No drug, light blue) or 2 µM veliparib (dark blue) for 24 hours. Linear regressions are shown for both datasets (n=2). **d**, Signal-to-background calculations across different cell numbers plated (from **c**). n=2 with corresponding linear regression. **e**, Cells (as in **a**) were plated in the absence (grey) or presence of DOX (blue) and exposed to DMSO or serial dilutions of olaparib for 24 hours. n=3 and relevant curve fitting are shown. **f**, Fold expression of *nLuc* in pooled HCT116 pCW57.1-PARP1 L713F-nLuc cells after normalization to *GAPDH* and *β-actin* by RT-qPCR. Means ± SD are shown (n=3). *P* values shown for comparisons by two-way ANOVA with multiple comparisons to "No DOX" control for each primer pair (Dunnett's test, F [DFn, DFd]: $F_{Interaction}$ [2, 12] = 6.517, $F_{Column\ Factor}$ [2, 12] =

89.70, $F_{Row\ Factor}$ [1, 12] = 5.544). **g**, After induction, HCT116 pCW57.1-PARP1 L713F-nLuc cells were treated with serial dilutions of talazoparib, 3-AB, or iniparib diluted in Fluorobrite DMEM (24 hours) before luminescence detection (open filter). n=2 and relevant curve fittings are shown. **i**, The same experiments from **h** but measured on a nLuc-specific filter. n=2 and relevant curve fittings are shown. **j**, HCT116 pLenti CMV Blast-PARP1 L713F-nLuc cells were treated with DMSO or veliparib for 24 hours before measuring luminescence (open filter). n=2 and relevant curve fittings are shown. RLU – relative luminescence units.

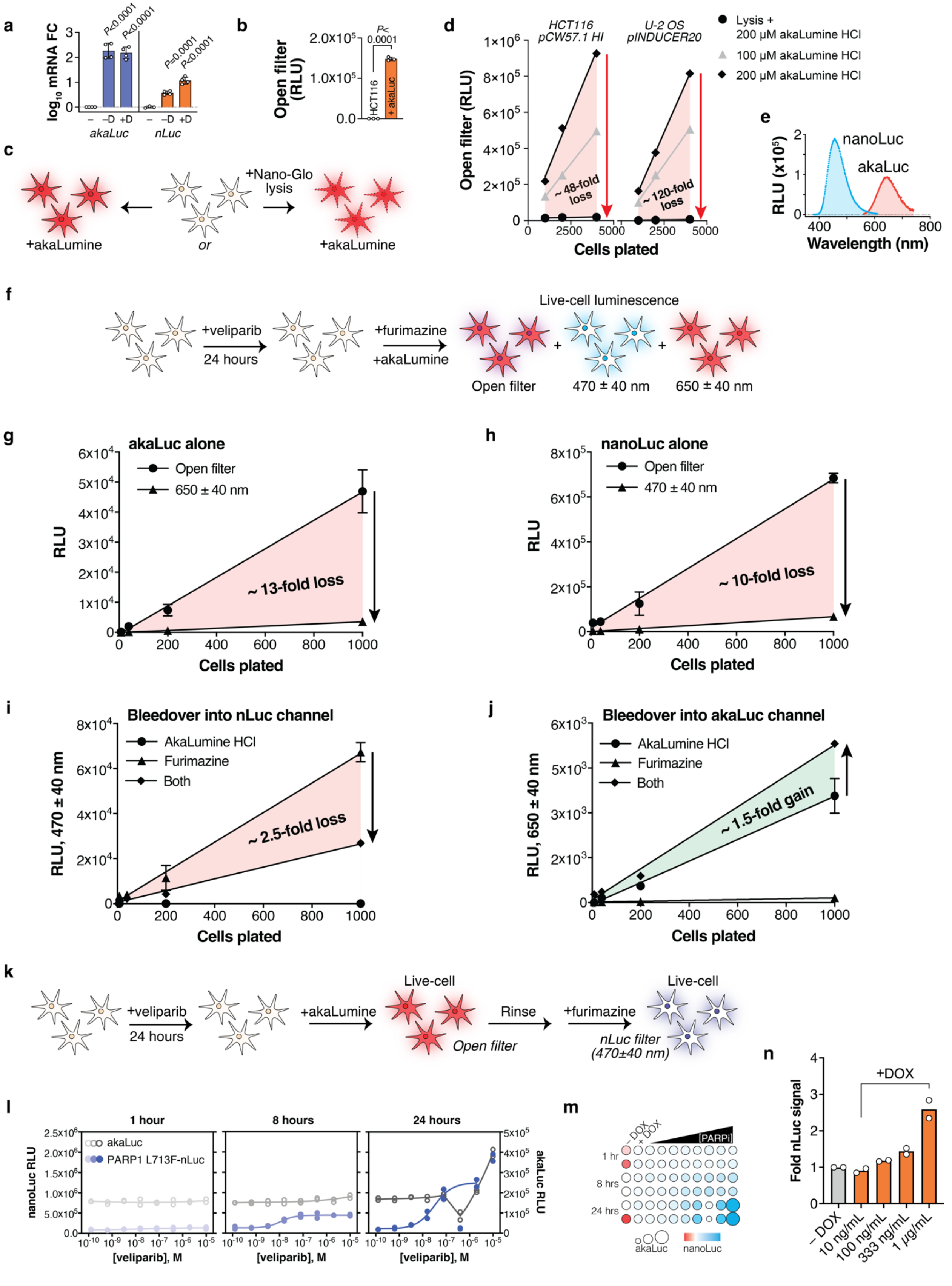
a
log10 mRNA FC
P<0.0001
P<0.0001
P<0.0001
P<0.0001
akaLuc
nLuc
b
Open filter (RLU)
P< 0.0001
HCT116
+ akaLuc
c
+Nano-Glo lysis
+akaLumine
or
+akaLumine
d
HCT116 pCW57.1 HI
U-2 OS pINDUCER20
Open filter (RLU)
~ 48-fold loss
~ 120-fold loss
Cells plated
Lysis + 200 µM akaLumine HCl
100 µM akaLumine HCl
200 µM akaLumine HCl
e
nanoLuc
akaLuc
RLU (x10^5)
Wavelength (nm)
f
+veliparib 24 hours
+furimazine +akaLumine
Live-cell luminescence
Open filter
470 ± 40 nm
650 ± 40 nm
g
akaLuc alone
Open filter
650 ± 40 nm
RLU
~ 13-fold loss
Cells plated
h
nanoLuc alone
Open filter
470 ± 40 nm
RLU
~ 10-fold loss
Cells plated
i
Bleedover into nLuc channel
AkaLumine HCl
Furimazine
Both
RLU, 470 ± 40 nm
~ 2.5-fold loss
Cells plated
j
Bleedover into akaLuc channel
AkaLumine HCl
Furimazine
Both
RLU, 650 ± 40 nm
~ 1.5-fold gain
Cells plated
k
+veliparib 24 hours
+akaLumine
Live-cell
Open filter
Rinse
+furimazine
Live-cell
nLuc filter (470±40 nm)
l
1 hour
8 hours
24 hours
akaLuc
PARP1 L713F-nLuc
nanoLuc RLU
akaLuc RLU
[veliparib], M
m
– DOX
+ DOX
[PARPi]
1 hr
8 hrs
24 hrs
akaLuc
nanoLuc
n
+DOX
Fold nLuc signal
– DOX
10 ng/mL
100 ng/mL
333 ng/mL
1 µg/mL

Supplementary Figure 17. AkaLuc suitability as an nLuc normalization factor. a, RT-qPCR analysis of *akaLuc* (blue) and *nLuc* (orange) expression in parental HCT116 (–; grey) or HCT116 pCW57.1-PARP1 L713F-nLuc/pLenti CMV Blast-akaLuc cells -/+DOX (24 hours) normalized to *GAPDH*/*β-actin*. Means of n=3 (parental HCT116) or n=4 (all others; duplicates) ± SD plotted in $\log_{10}$ scale. **b**, Cells were treated as in **a**, then measured (open filter) after 200 µM akaLumine HCl. Means of n=3 (duplicates) ± SD. **c**, akaLuc amenability to lytic conditions procedure. **d**, Induced HCT116 pCW57.1-PARP1 L713F-nLuc/pLenti CMV Blast-akaLuc or U-2 OS pINDUCER20-PARP1 L713F-nLuc/pLenti-CMV-blast-akaLuc cells plated at different densities after 200 µM akaLumine HCl in Nano-Glo lysis reagent or 100-200 µM in FluoroBrite DMEM (each n=1). **e**, Emission and intensity spectra of L713F-nLuc (blue) and akaLuc (red). **f**, Concomitant nLuc/akaLuc readout procedure. **g**, akaLuc detection by open or akaLuc-specific filter, as in **f**. **h**, L713F-nLuc detection by open or nLuc-specific filter, as in **f**. **i**, Comparison of nLuc filter signal with akaLumine HCl, furimazine, or both simultaneously. **j**, Comparison of akaLuc filter signal, as in **i**. For **g-j**, means of n=2 ± range and estimated luminescence intensity changes are shown. **k**, Sequential akaLuc/L713F-nLuc readout procedure in live cells. **l**, Time-dependent maturation of L713F-nLuc (left axis, blue) compared to akaLuc (right axis, grey) after veliparib treatment. A single experiment (duplicates) is shown. **m**, Normalization matrix relating L713F-nLuc signal to akaLuc signal with data from **l**. Circle size–akaLuc intensity (65,451 to 414,241 RLU); red-white-blue gradient–nLuc intensity (73,523 to 1,954,226 RLU). **n**, Cells were incubated with the indicated concentration of doxycycline (24 hours) before sequential akaLuc-nLuc measurement. Mean nLuc/akaLuc fold change relative to "– DOX" shown from n=2 experiments (subset of data from **Fig. 6c**). *P* values shown for comparisons by one-way ANOVA analysis with multiple comparisons to parental HCT116 cells for each primer set (Dunnett's test; [**a**; $F_{\text{Treatment akaLuc}}$ (DFn, DFd) = 130.0 (2, 9), $F_{\text{Treatment nLuc}}$ (DFn, DFd) = 97.61 (2, 8)] or unpaired, two-tailed t test (**b**; t, df = 59.72, 4). FC – fold change; RLU – relative luminescence units.

a

Cell line/vector	Z'	S/B
HCT116 pCW57.1	0.51	11.19
U-2 OS pINDUCER	0.77	6.47
HCT116 pLenti	0.83	5.88

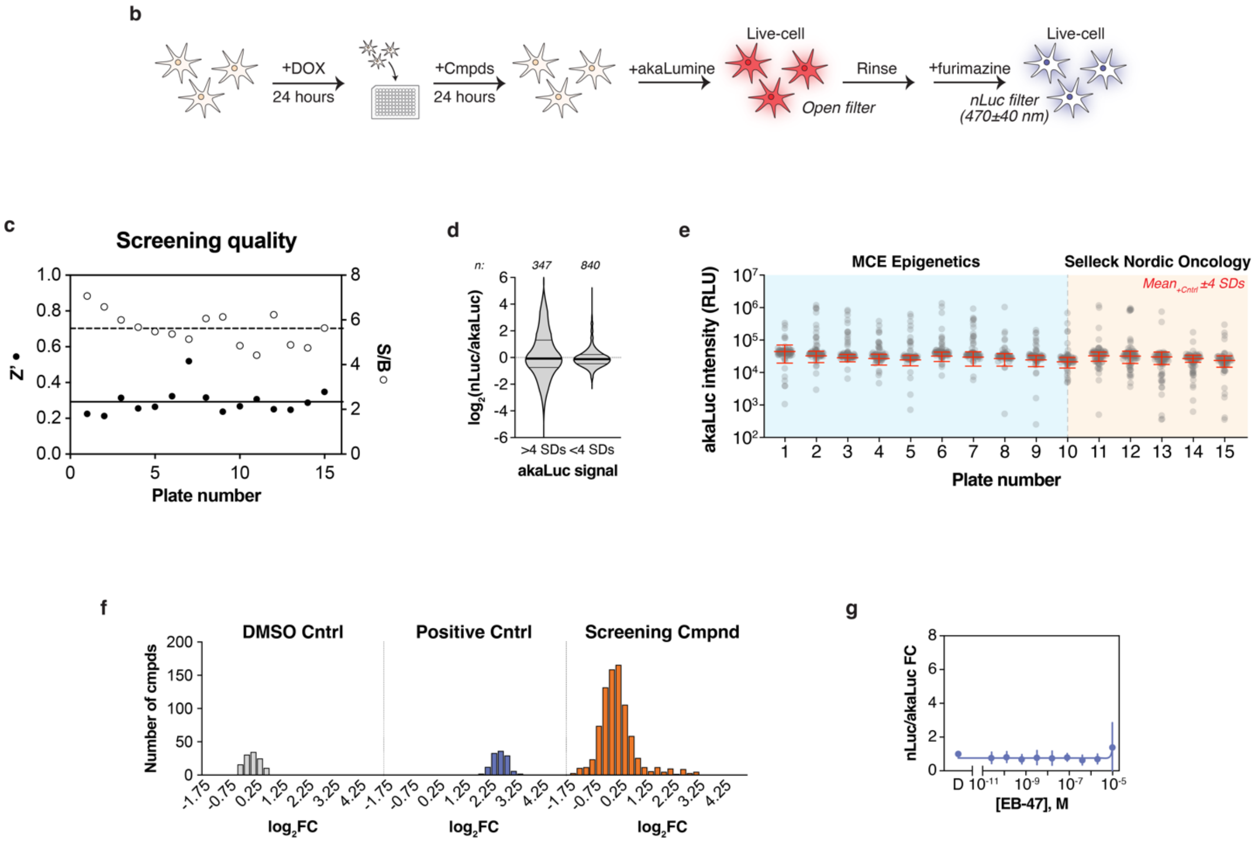

Supplementary Figure 18. Data quality and analysis from PARP1 L713F biophysical perturbagen screen. a, A comparison of Z' and signal-to-background (S/B) values across cell and expression systems from earlier testing. Z' = 1 – [$3\sigma_+ + 3\sigma_-$] / |$\mu_+ - \mu_-$| and S/B = [mean signal] / [mean background], as defined by Zhang, Chung, & Oldenburg[6]. **b**, Screening procedure overview. **c**, Screening quality summary on a per plate basis looking at Z' (left axis, closed circles) and S/B (right axis, open circles) based on the positive and negative controls on each plate with average values indicated. **d**, Violin plots of $\log_2$-transformed nLuc/akaLuc ratios for the screening library triaged by akaLuc signal being within (right) or beyond four SDs (left) of controls. Medians (thick lines) and quartiles (thin lines), as well as the number of screening compounds falling into each category are shown. **e**, Per-plate scatter plots of akaLuc intensity from screening compounds with overlayed means ± 4 SDs from positive controls (red). Library designations are also labeled (MCE Epigenetics – blue; Selleck Nordic Oncology – orange). **f**, Histograms showing improved normality of $\log_2$-transformed nLuc/akaLuc ratios for DMSO control (negative; grey), veliparib control (positive; blue), and screening library compounds (orange). Bin width – 0.25. **g**, Follow-up dose-response analysis with L713F-nLuc and EB-47. Means from n=3 (duplicates) ± SD and relevant curve fittings are shown. RLU – relative luminescence unit, FC – fold change. See Source Data file for more information.

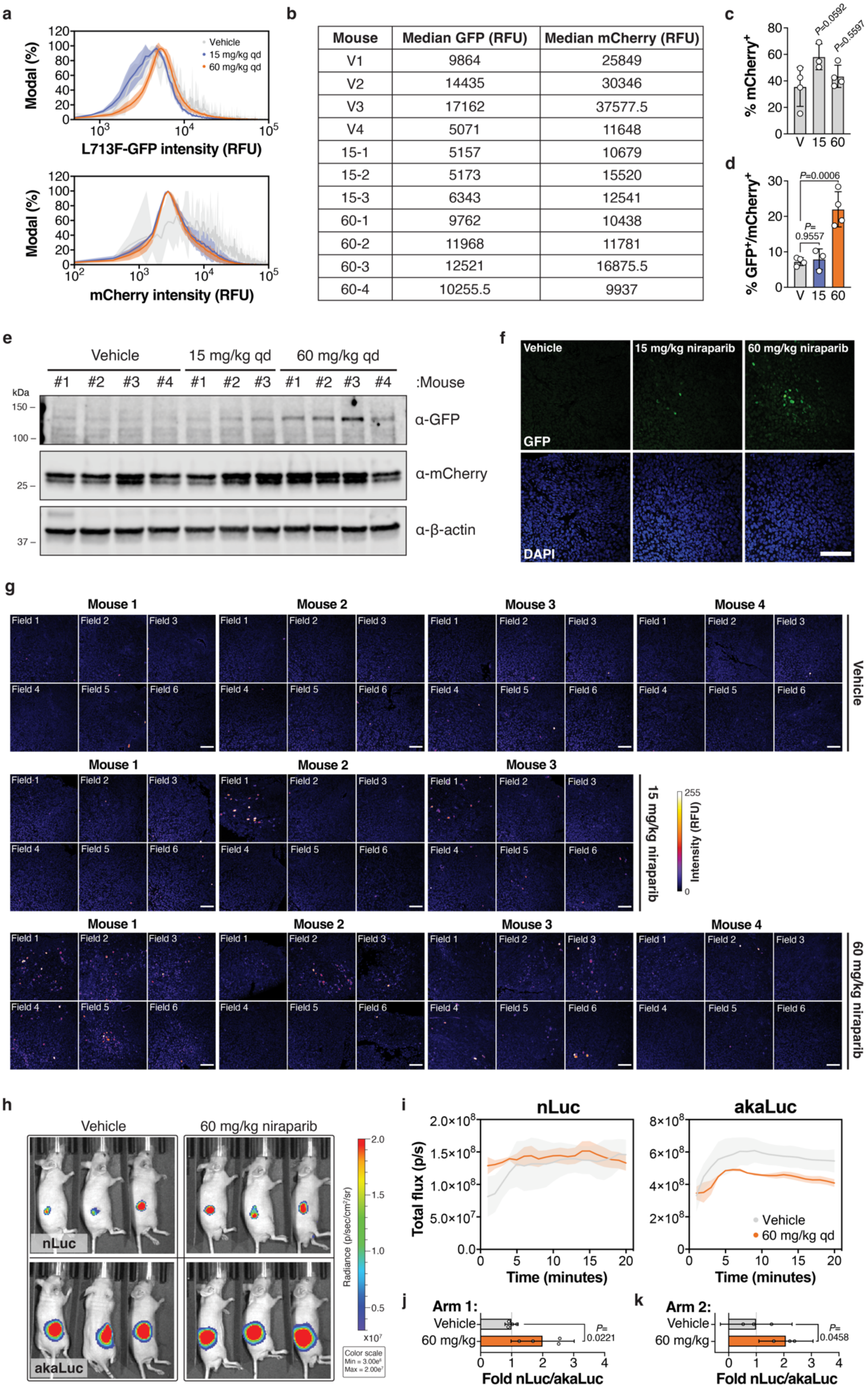

Mouse	Median GFP (RFU)	Median mCherry (RFU)
V1	9864	25849
V2	14435	30346
V3	17162	37577.5
V4	5071	11648
15-1	5157	10679
15-2	5173	15520
15-3	6343	12541
60-1	9762	10438
60-2	11968	11781
60-3	12521	16875.5
60-4	10255.5	9937

Supplementary Figure 19. Supporting data for *in vivo* CeTEAM analyses. a, Average L713F-GFP and mCherry intensity histograms from flow cytometry of live vehicle- (grey), 15 mg/kg- (blue), and 60 mg/kg-treated (orange) HCT116 tumor cells. Histograms are normalized to the mode for each dataset. The error bands (shading) denote the data ranges from $n_{Vehicle} = 4$, $n_{15mg/kg} = 3$, and $n_{60mg/kg} = 4$ mice. **b**, Median L713F-GFP and mCherry fluorescence intensities from *ex vivo* tumor cells by flow cytometry. **c**, Percent of live tumor cells identified as mCherry positive ($mCherry^{+}$). Means from individual mice ± SD are shown. **d**, Percent of live tumor cells identified as GFP/mCherry double positive ($GFP^{+}/mCherry^{+}$). Means from individual mice ± SD are shown. *P* values shown for comparisons by ordinary one-way ANOVA with multiple comparisons to the DMSO control (Dunnett's test; $F_{Treatment\ (\mathbf{c})}$ [DFn, DFd] = 3.334 [2, 8], $F_{Treatment\ (\mathbf{d})}$ [DFn, DFd] = 22.60 [2, 8]). **e**, Full western blot from **Fig. 7g**, including mCherry and β-actin loading controls. **f**, anti-GFP staining of representative PARP1 L713F-GFP/mCherry tumor sections from vehicle- (mouse 1, field 6), 15 mg/kg- (mouse 3, field 5), or 60 mg/kg niraparib-treated mice (mouse 2, field 5) counterstained with DAPI. Scale bar=100 µm. **g**, Micrographs of L713F-GFP saturation in tumor sections by fire LUT. Six fields taken per section. **h**, Representative bioluminescence overlays for L713F-nLuc and akaLuc radiance from experimental arm 2 (n=3 mice per group). **i**, Change in total luminescent flux (photons/second) over 20 minutes for both L713F-nLuc and akaLuc following substrate injection in mice treated with vehicle (grey) or 60 mg/kg niraparib (orange). n=3 mice per group (arm 2) ± SEM. **j**, Comparison of the mean fold change in L713F-nLuc/akaLuc signal from experimental arm 1 (n=4 mice per group) and arm 2 (**k**, n=3 mice per group). In **j** and **k**, error denotes 95% confidence interval, and *P* values shown for comparisons by two-tailed, unpaired t test (t, df = $[3.064, 6]_{\mathbf{h}}$, $[2.863, 4]_{\mathbf{i}}$). RFU – relative fluorescence units.

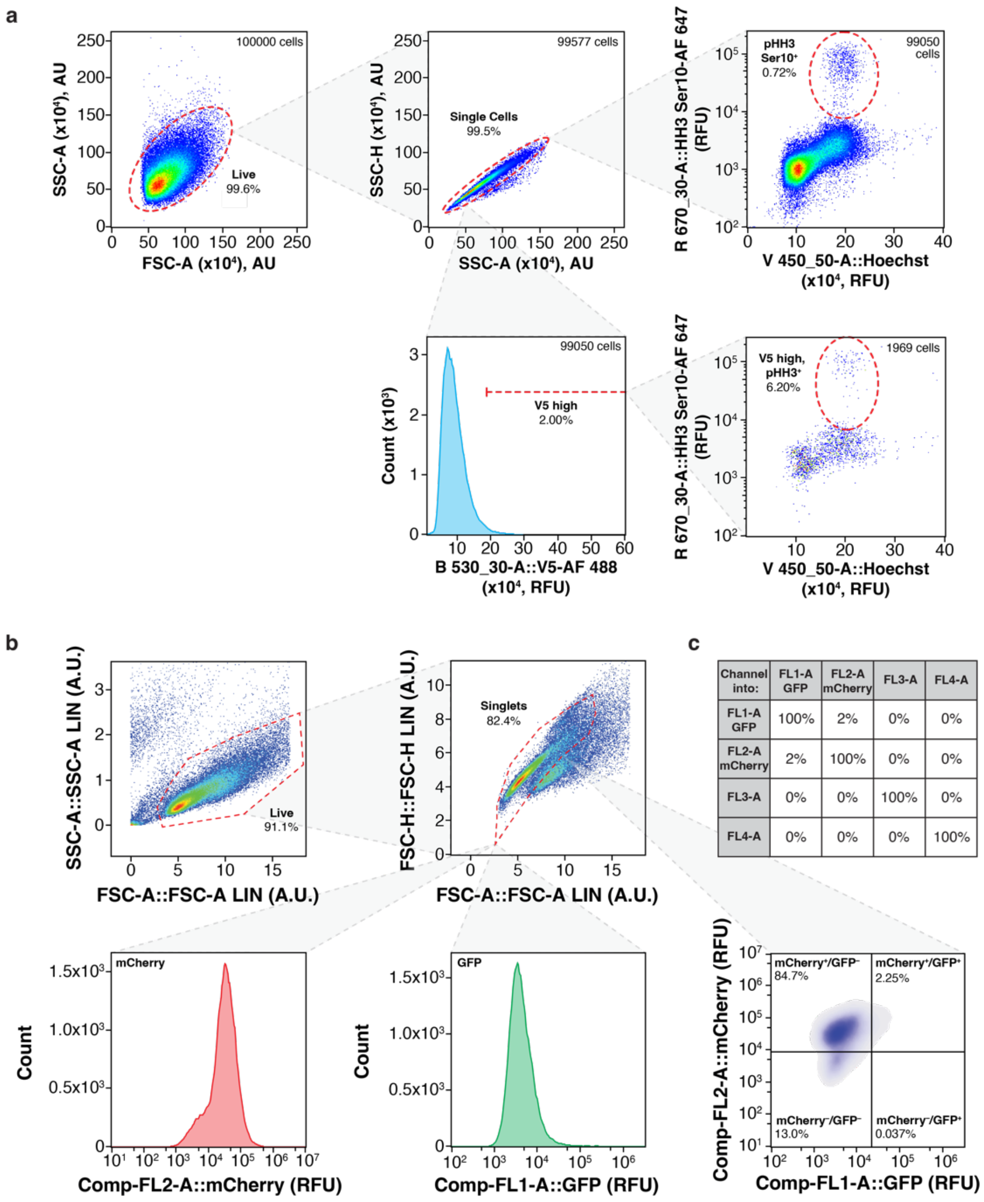

Channel into:	FL1-A GFP	FL2-A mCherry	FL3-A	FL4-A
FL1-A GFP	100%	2%	0%	0%
FL2-A mCherry	2%	100%	0%	0%
FL3-A	0%	0%	100%	0%
FL4-A	0%	0%	0%	100%

Supplementary Figure 20. Gating strategies used for flow cytometry analyses. a, Gating of V5-MTH1 G48E cells. "Live cells" were identified and gated to identify "Single Cells" before identification of "pHH3 Ser10^{+}" cells. "Single cells" were gated to identify "V5 high" cells (arbitrarily defined as top 2% of V5 signal in the DMSO control), from which "V5 high/pHH3^{+}" cells were identified. **b**, PARP L713F GFP/mCherry gating strategy. "Live cells" were identified and gated as "singlet" cells before identification of "mCherry^{+}" and "GFP^{+}" cells. This population was also plotted in two dimensions to simultaneously follow mCherry and GFP signals and to establish compensation parameters (**c**). The examples shown are for DMSO-treated cells but applies identically to cells incubated with inhibitors *in vitro* or *in vivo*. Axes are denoted with relevant laser, filter, marker, and fluorescent label. RFU – relative fluorescence units.

Supplementary References

1. Fakan, S., Leduc, Y., Lamarre, D., Brunet, G. & Poirier, G.G. Immunoelectron microscopical distribution of poly(ADP-ribose)polymerase in the mammalian cell nucleus. *Exp Cell Res* **179**, 517-526 (1988).
2. Engbrecht, M. & Mangerich, A. The Nucleolus and PARP1 in Cancer Biology. *Cancers (Basel)* **12** (2020).
3. Haince, J.F. et al. PARP1-dependent kinetics of recruitment of MRE11 and NBS1 proteins to multiple DNA damage sites. *J Biol Chem* **283**, 1197-1208 (2008).
4. Rank, L. et al. Analyzing structure-function relationships of artificial and cancer-associated PARP1 variants by reconstituting TALEN-generated HeLa PARP1 knock-out cells. *Nucleic Acids Res* **44**, 10386-10405 (2016).
5. Zandarashvili, L. et al. Structural basis for allosteric PARP-1 retention on DNA breaks. *Science* **368** (2020).
6. Zhang, J.H., Chung, T.D. & Oldenburg, K.R. A Simple Statistical Parameter for Use in Evaluation and Validation of High Throughput Screening Assays. *J Biomol Screen* **4**, 67-73 (1999).

.ta File Z:\2016\1608BR\3399.D
.mple Name: BZ6672

TH8228
LCMS

Acq. Operator	: Brutus_br	Seq. Line :	1
Acq. Instrument	: Instrument 1	Location :	Vial 88
Injection Date	: 8/15/2016 11:14:50 AM	Inj :	1
		Inj Volume :	2 µl

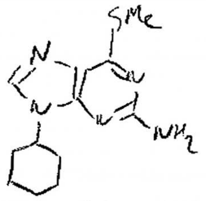

(263.4)

Sequence File : D:\SEQUENCE\USETHIS.S
Method : D:\METHODS\STANDARD_B1090A.M
Last changed : 8/15/2016 11:13:45 AM by Brutus_br
(modified after loading)
Method Info : 10-90% MeCN-1.5min, ACE C8, 50x3.0 mm, 3u, 1ml/min, 220-395, 254, 220 nm,
A: 0.1% TFA, B: MeCN

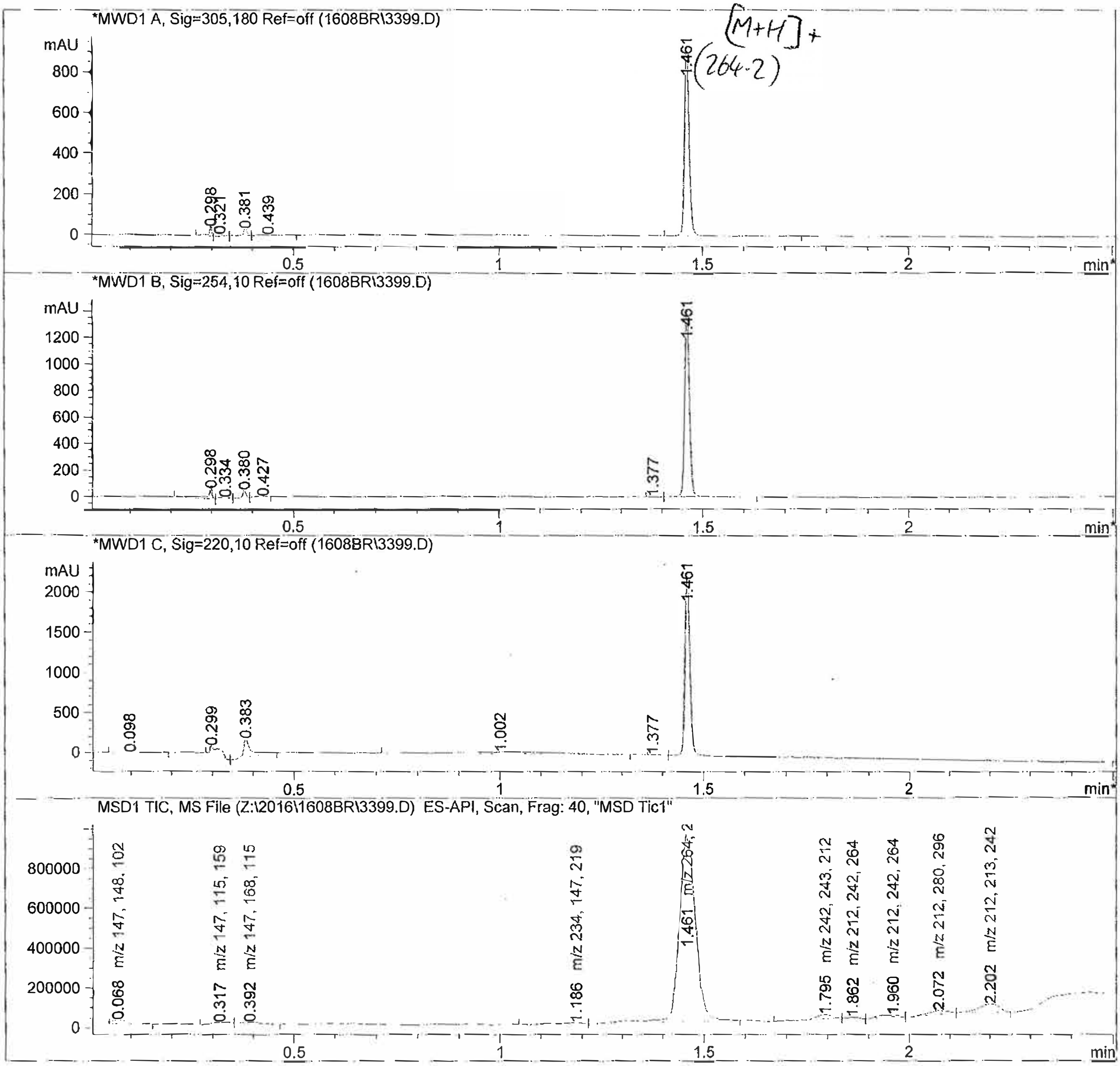

TH8228 ^{1}H + ^{13}C NMR

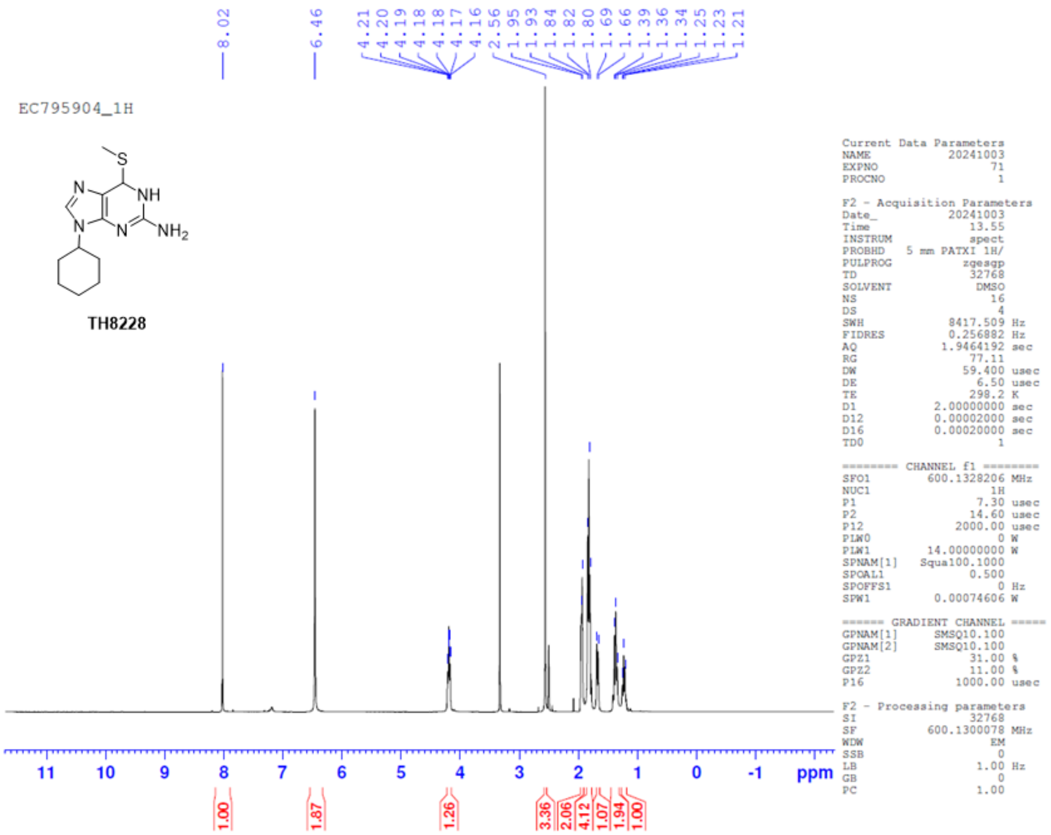

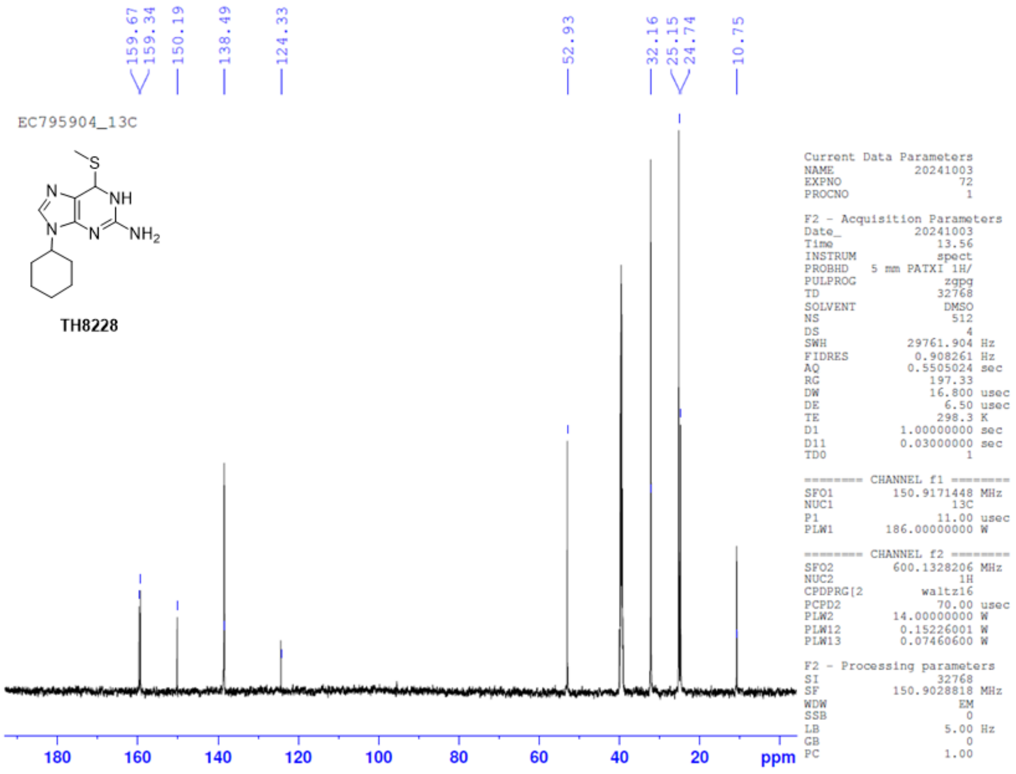

a File Z:\2016\1608BR\3402.D
nple Name: BZ6680

TH8234
LCMS

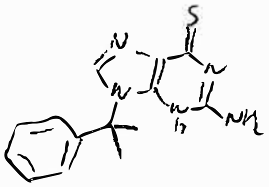

Acq. Operator	: Brutus_br		Seq. Line	:	9
Acq. Instrument	: Instrument 1		Location	:	Vial 78
Injection Date	: 8/15/2016 11:54:13 AM		Inj	:	1
			Inj Volume	:	2 µl

Sequence File : D:\SEQUENCE\USETHIS.S
Method : D:\METHODS\STANDARD_B1090A.M
Last changed : 8/15/2016 11:48:19 AM by Brutus_br
(modified after loading)
Method Info : 10-90% MeCN-1.5min, ACE C8, 50x3.0 mm, 3u, 1ml/min, 220-395, 254, 220 nm,
A: 0.1% TFA, B: MeCN

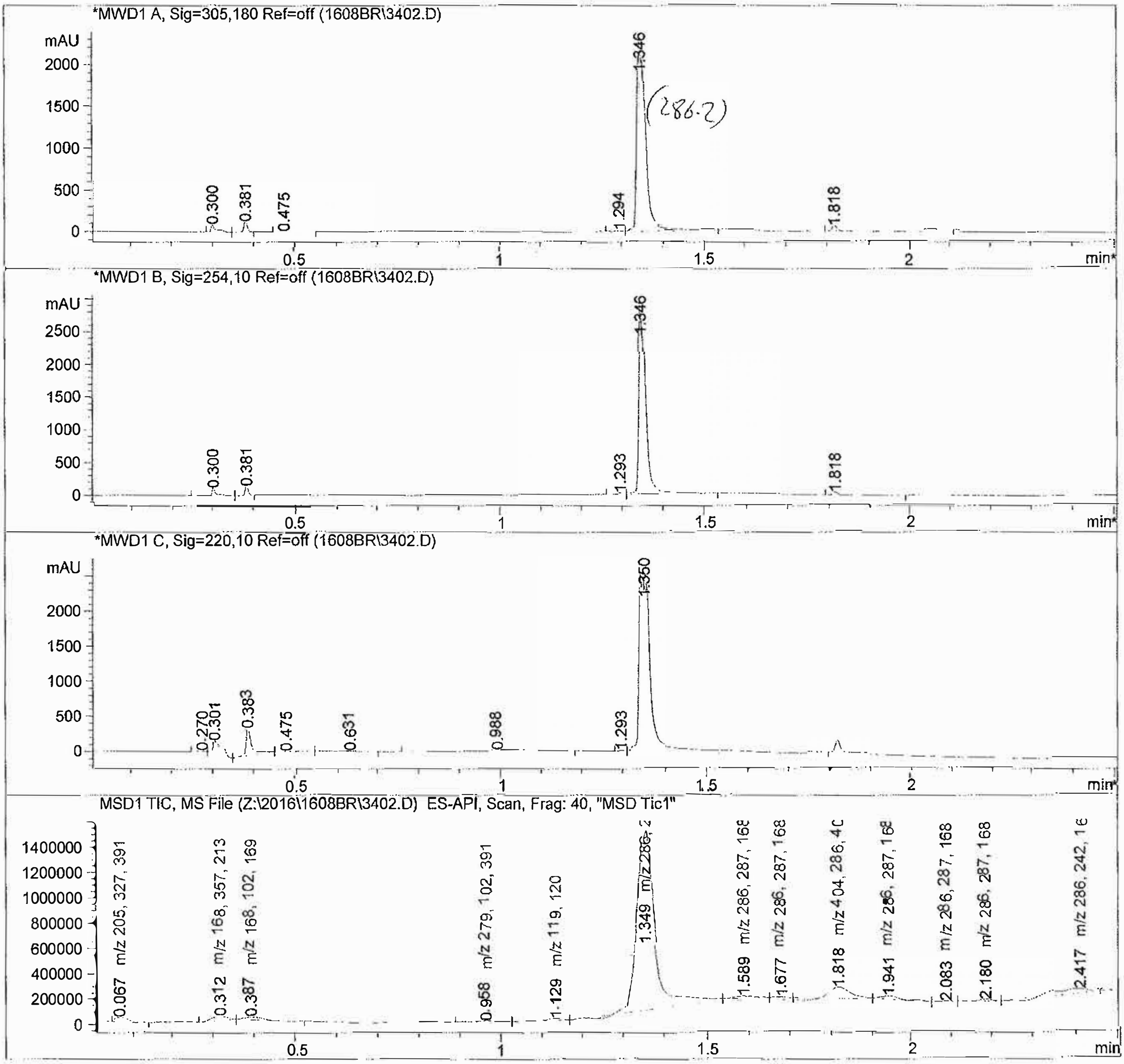

TH8234 ^{1}H + ^{13}C NMR

^{1}H NMR thioamide tautomer

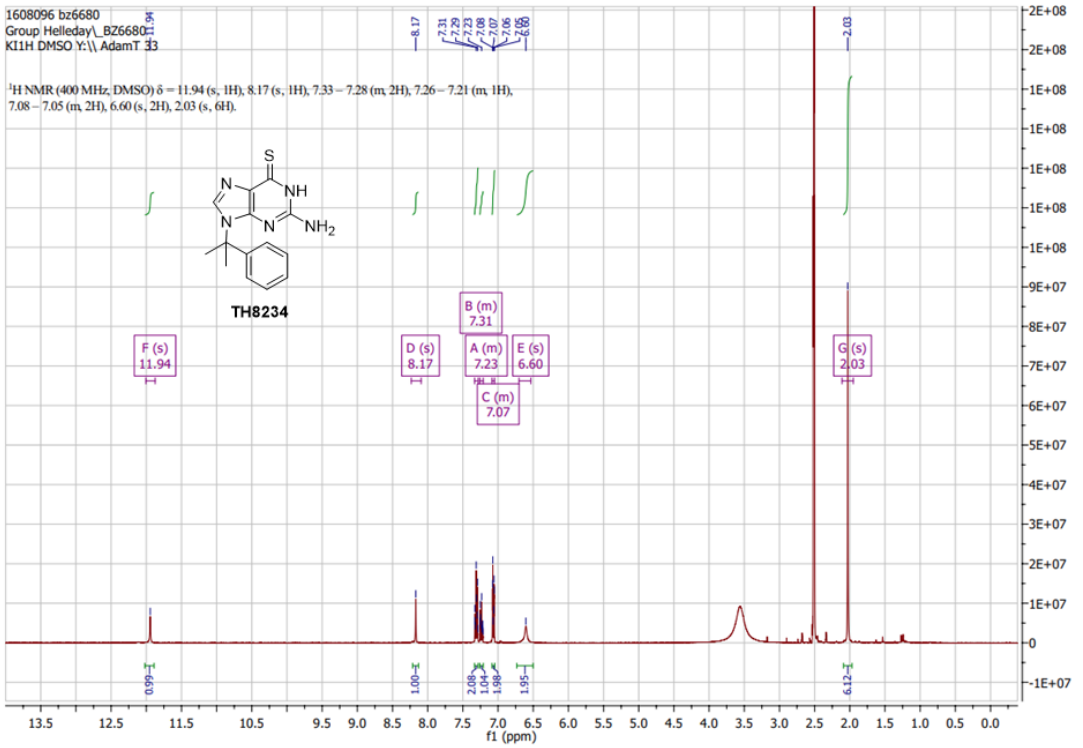

TH8234 ^{1}H and ^{13}C NMRs mixture of thioamide + iminothiol tautomers

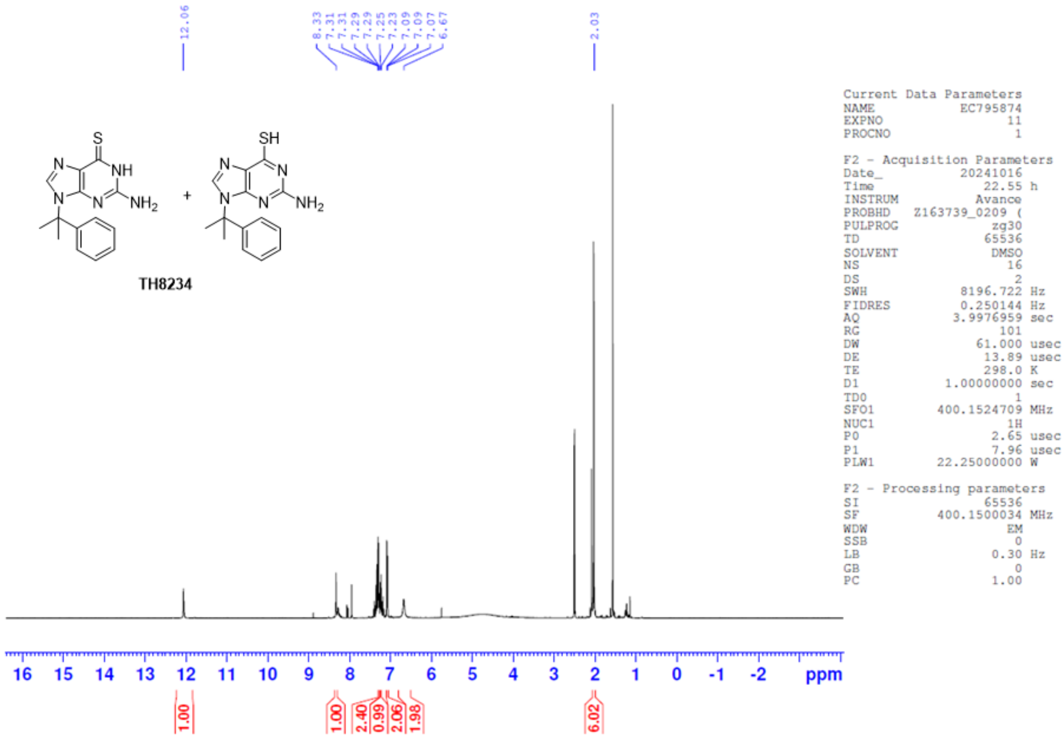

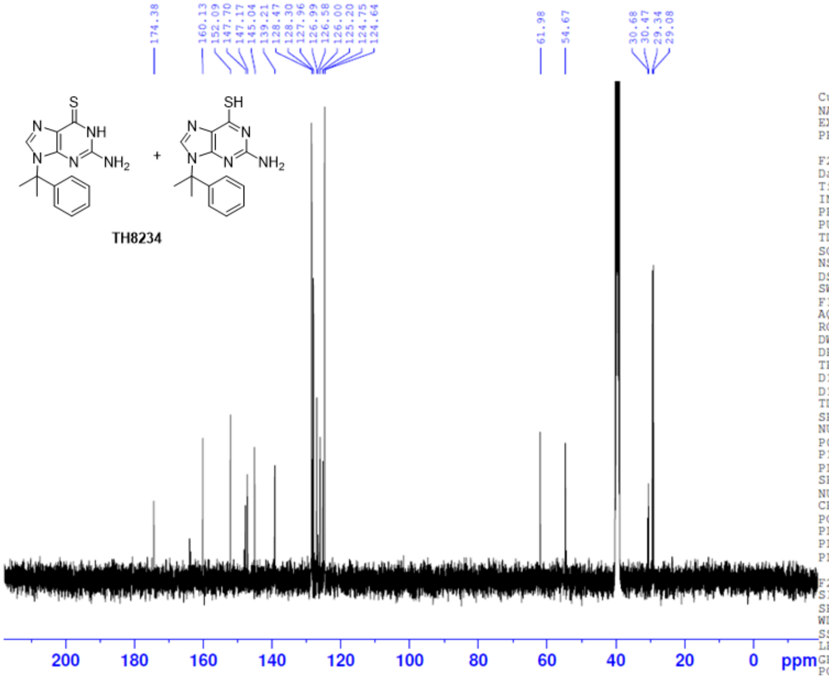


```
Current Data Parameters
NAME              EC795874
EXPNO                   12
PROCNO                   1

F2 - Acquisition Parameters
Date_             20241016
Time                 23.55 h
INSTRUM             Avance
PROBHD    Z163739_0209 (
PULPROG            zgpg30
TD                  65536
SOLVENT              DMSO
NS                   1024
DS                      4
SWH             23809.523 Hz
FIDRES           0.726609 Hz
AQ              1.3762560 sec
RG                    101
DW                 21.000 usec
DE                   6.50 usec
TE                  298.0 K
D1             2.00000000 sec
D11            0.03000000 sec
TD0                     1
SFO1          100.6278593 MHz
NUC1                  13C
P0                   2.67 usec
P1                   8.00 usec
PLW1          95.14800262 W
SFO2          400.1516006 MHz
NUC2                   1H
CPDPRG[2          waltz65
PCPD2               90.00 usec
PLW2          22.25000000 W
PLW12          0.17405000 W
PLW13          0.08754500 W

F2 - Processing parameters
SI                  32768
SF            100.6178455 MHz
WDW                    EM
SSB                     0
LB                   1.00 Hz
GB                      0
PC                   1.40
```
