# Supplementary material for: Coupling cellular drug-target engagement to downstream pharmacology with CeTEAM
Source: Nat Commun. 2024 Dec 6;15:10347. doi: 10.1038/s41467-024-54415-7 (PMC11624193; doi:10.1038/s41467-024-54415-7)
Supplement: Supplementary file 2 — Description of Additional Supplementary Files [file 41467_2024_54415_MOESM2_ESM.pdf]

Description of Additional Supplementary Files

Supplementary Data 1 – Screening compounds and results for potential modulators of PARP1 L713F-nLuc abundance.

Supplementary Data 2 – A summary of all plasmids, primers, and synthetic gene fragments used in this study. Sequences are provided for all primers, gene fragments, and custom vectors.
