# Supplementary material for: Coupling cellular drug-target engagement to downstream pharmacology with CeTEAM
Source: Nat Commun. 2024 Dec 6;15:10347. doi: 10.1038/s41467-024-54415-7 (PMC11624193; doi:10.1038/s41467-024-54415-7)
Supplement: Supplementary file 5 — Reporting Summary [file 41467_2024_54415_MOESM5_ESM.pdf]

nature portfolio

Corresponding author(s): Nicholas Valerie

Last updated by author(s): Jul 31, 2024

Reporting Summary

Nature Portfolio wishes to improve the reproducibility of the work that we publish. This form provides structure for consistency and transparency in reporting. For further information on Nature Portfolio policies, see our Editorial Policies and the Editorial Policy Checklist.

Statistics

For all statistical analyses, confirm that the following items are present in the figure legend, table legend, main text, or Methods section.

n/a	Confirmed	
☐	☒	The exact sample size (n) for each experimental group/condition, given as a discrete number and unit of measurement
☐	☒	A statement on whether measurements were taken from distinct samples or whether the same sample was measured repeatedly
☐	☒	The statistical test(s) used AND whether they are one- or two-sided<br>*Only common tests should be described solely by name; describe more complex techniques in the Methods section.*
☒	☐	A description of all covariates tested
☐	☒	A description of any assumptions or corrections, such as tests of normality and adjustment for multiple comparisons
☐	☒	A full description of the statistical parameters including central tendency (e.g. means) or other basic estimates (e.g. regression coefficient) AND variation (e.g. standard deviation) or associated estimates of uncertainty (e.g. confidence intervals)
☐	☒	For null hypothesis testing, the test statistic (e.g. F, t, r) with confidence intervals, effect sizes, degrees of freedom and P value noted<br>*Give P values as exact values whenever suitable.*
☒	☐	For Bayesian analysis, information on the choice of priors and Markov chain Monte Carlo settings
☒	☐	For hierarchical and complex designs, identification of the appropriate level for tests and full reporting of outcomes
☒	☐	Estimates of effect sizes (e.g. Cohen's d, Pearson's r), indicating how they were calculated

Our web collection on statistics for biologists contains articles on many of the points above.

Software and code

Policy information about availability of computer code

Data collection	CFX96 Real-Time PCR machine, CFX Maestro™ 1.0 Software, version 4.0.2325.0418 (Bio-Rad); Lightcycler 480 Software, version 1.5.1.62 (Roche); ZOE Cell Image Software version: 2.257 (Bio-Rad); EVOS® FL Cell Imaging System Software, version 1.4 (Thermo Fisher Sciencitific); MetaXpress High-Content Image Acquisition and Analysis Software, version 5 (Molecular Devices); Hidex Sense Platereader Software, version 0.5.11.2 (Aurentia Solutions); CLARIOstar software, version 5.40 R2 (BMG LABTECH); Image Studio, version 5.2 (Li-Cor Biosciences); BD Accuri C6 Software, version 1.0.264.21 (Bectin Dickenson); ZEN Software, version 2.1 (ZEISS); Fiji, version 2.1.0/1.53c (ImageJ); Living Image Software, version 4.7.2 (Perkin Elmer); CELLCYTE Studio, version 2.7.4 (CYTENA); BD FACSDiva software, version 8.0.1 (Bectin Dickenson)
Data analysis	Microsoft Excel 2010 (Microsoft); Prism, version 10 (GraphPad Software); ImageStudio Lite, version 5.2.5 (Li-Cor Biosciences); Kaluza Analysis software (version 2.1.00000.20651); FlowJo, version 10.7.1 (Bectin Dickenson); CellProfiler, version 3.1.0 (Broad Institute); Fiji, version 2.1.0/1.53c (ImageJ); iMOSFLM, version 7.2.1 (Battye et al., 2011, Acta Crystallogr. D); DIALS, version 2.0 from the CCP4 suite (Winter et al., Acta Crystallogr. Sect. D, Struct Biol); Aimless, version 0.5.7 from the CCP4 suite, version 6.5.020 (Collaborative Computational Project, 2011, Acta Crystallogr. D); Refine and eLBOW from the Phenix suite, version 1.14 (Liebschner et al., 2019, Acta Crystallogr. D); Phaser, version 2.8.2 (McCoy et al., 2007, J. Appl. Crystallogr.); TLSMD webserver (Painter et al., 2006, Acta Crystallogr. D); PDB_REDO webserver (Joosten et al., 2014, IUCrJ); CLARIOstar MARS Software, version 3.31 (BMG LABTECH).

For manuscripts utilizing custom algorithms or software that are central to the research but not yet described in published literature, software must be made available to editors and reviewers. We strongly encourage code deposition in a community repository (e.g. GitHub). See the Nature Portfolio guidelines for submitting code & software for further information.

Data

Policy information about availability of data

All manuscripts must include a data availability statement. This statement should provide the following information, where applicable:

- Accession codes, unique identifiers, or web links for publicly available datasets
- A description of any restrictions on data availability
- For clinical datasets or third party data, please ensure that the statement adheres to our policy

The data generated in this study is available in the main text, Supplementary Information, or Source Data file (provided with this paper). The NSC56456-NUDT15 co-crystallization data generated in this study has been deposited in the RCSB Protein Data Bank database under accession code 7NR6 [https://doi.org/10.2210/pdb7NR6/pdb]. PDB accession codes 7KK2 [https://doi.org/10.2210/pdb7KK2/pdb] and 3KCZ [https://doi.org/10.2210/pdb3KCZ/pdb] were previously published. Materials are available from the corresponding author upon request.

Research involving human participants, their data, or biological material

Policy information about studies with human participants or human data. See also policy information about sex, gender (identity/presentation), and sexual orientation and race, ethnicity and racism.

Reporting on sex and gender

Use the terms sex (biological attribute) and gender (shaped by social and cultural circumstances) carefully in order to avoid confusing both terms. Indicate if findings apply to only one sex or gender; describe whether sex and gender were considered in study design; whether sex and/or gender was determined based on self-reporting or assigned and methods used.
Provide in the source data disaggregated sex and gender data, where this information has been collected, and if consent has been obtained for sharing of individual-level data; provide overall numbers in this Reporting Summary. Please state if this information has not been collected.
Report sex- and gender-based analyses where performed, justify reasons for lack of sex- and gender-based analysis.

Reporting on race, ethnicity, or other socially relevant groupings

Please specify the socially constructed or socially relevant categorization variable(s) used in your manuscript and explain why they were used. Please note that such variables should not be used as proxies for other socially constructed/relevant variables (for example, race or ethnicity should not be used as a proxy for socioeconomic status).
Provide clear definitions of the relevant terms used, how they were provided (by the participants/respondents, the researchers, or third parties), and the method(s) used to classify people into the different categories (e.g. self-report, census or administrative data, social media data, etc.)
Please provide details about how you controlled for confounding variables in your analyses.

Population characteristics

Describe the covariate-relevant population characteristics of the human research participants (e.g. age, genotypic information, past and current diagnosis and treatment categories). If you filled out the behavioural & social sciences study design questions and have nothing to add here, write "See above."

Recruitment

Describe how participants were recruited. Outline any potential self-selection bias or other biases that may be present and how these are likely to impact results.

Ethics oversight

Identify the organization(s) that approved the study protocol.

Note that full information on the approval of the study protocol must also be provided in the manuscript.

Field-specific reporting

Please select the one below that is the best fit for your research. If you are not sure, read the appropriate sections before making your selection.

☒ Life sciences ☐ Behavioural & social sciences ☐ Ecological, evolutionary & environmental sciences

For a reference copy of the document with all sections, see nature.com/documents/nr-reporting-summary-flat.pdf

Life sciences study design

All studies must disclose on these points even when the disclosure is negative.

Sample size

No sample-size calculations were performed. Sample sizes for flow cytometry experiments (number of events) were at least 20,000 singlet events (100,000 for low-abundance pHH3 Ser10 populations) to guarantee sufficient numbers for e.g., cell cycle analysis based on guidelines set forth by Cossarizza et al., 2019. Guidelines for the use of flow cytometry and cell sorting in immunological studies (second edition). Eur J Immunol; 49(10):1457-1973. For general microscopy experiments, 750-4,000 cells were plated depending on the length of the experiment and treatments studied. All cells within the field were counted and measured in an unbiased manner using an automated CellProfiler pipeline to routinely yield 500-2,000 cells per sample, similar to earlier work (Page & Valerie, "Targeted NUDT5 inhibitors block hormone signaling in breast cancer cells". Nature Comms, 2018). Collection from 20 (microirradiation recruitment experiments) and 50 individual cells (FRAP) are also similar, if not more samples, than similar studies (Xie et al., "Timeless interacts with PARP-1 to promote homologous recombination repair.", Molecular Cell, 2015). For animal work, our standard number of mice was ≥4 per group, which we believed would be sufficient to ensure statistical significance and account for higher variability, while maintaining ethical compliance by limiting the number of animals used in the study. This sample size is consistent with similar publications evaluating fluorescent or bioluminescent signals in animals, such as Su et al., "Novel NanoLuc substrates enable bright two-population bioluminescence imaging in animals", Nature Methods, 2020. For non-invasive

	luminescence studies, 7 animals were used per group but were then subdivided into two arms due to differences in xenografted tumor growth rate. This was to ensure that we could maximize signal from the experiment and comply with ethical standards.
Data exclusions	No data were excluded from analysis.
Replication	Most attempts at replication were successful and are presented in the paper as part of the main or supplementary figures. In the case of the screening confirmation experiment, one of the duplicate plates had to be discarded due to contamination, so for some compounds only three replicates were performed instead of six.
Randomization	Tumor-bearing mice were randomly allocated to treatment groups, although they were subdivided based on xenograft tumor size for earlier or later drug treatment, as outlined above. As our endpoint can be normalized to tumor size (proportional signal from akaLuc reference), differences in actual tumor sizes do not influence results. Anti-tumor efficacy of our treatments were not considered as an endpoint.<br><br>For in vitro experiments, findings were replicated in several formats and by different researchers to minimize potential experimental bias.
Blinding	Blinding was not generally used for any in vitro or in vivo data collection or analysis, and, in many instances, not possible because of experimental set-up. However, quantification of microscopy images was performed with pre-set CellProfiler pipelines to standardize conditions and remove potential human bias, and flow cytometry analysis was performed in FlowJo using batch workflows based on control datasets to limit biases from experimental results. Similarly, relevant controls were used to set thresholds for e.g. flow cytometry gating and as standards for evaluating responses to experimental stimuli.

Reporting for specific materials, systems and methods

We require information from authors about some types of materials, experimental systems and methods used in many studies. Here, indicate whether each material, system or method listed is relevant to your study. If you are not sure if a list item applies to your research, read the appropriate section before selecting a response.

Materials & experimental systems

n/a	Involved in the study	
☐	☒	Antibodies
☐	☒	Eukaryotic cell lines
☒	☐	Palaeontology and archaeology
☐	☒	Animals and other organisms
☒	☐	Clinical data
☒	☐	Dual use research of concern
☒	☐	Plants

Methods

n/a	Involved in the study	
☒	☐	ChIP-seq
☐	☒	Flow cytometry
☒	☐	MRI-based neuroimaging

Antibodies

Antibodies used	anti-HA probe (mouse, clone F-7, cat. #sc7392, lot #L1281), anti-GFP (rabbit, cat. #sc8334, lot #D1907), anti-GFP (mouse, clone B-2, cat. #sc9996, lot #H2018), anti-PARP1 (mouse, clone F-2, cat. #sc8007, lot #D3019), and anti-SOD1 (mouse, clone G-11, cat. #sc17767, lot #G3119) were obtained from Santa Cruz Biotechnology. anti-CHK1 (mouse, clone 2G1D5, cat. #2360S, lot #8), anti-p-CHK1 Ser345 (rabbit polyclonal, cat. #2341S, lot #8), anti-p-CHK1 Ser345 (rabbit, clone 133D3, cat. #2348S, lot #18), anti-p-Histone H2A.X Ser139 (H2A.X, rabbit, cat. #2577S, lot #12), and anti-vinculin (rabbit, cat. #4650S, lot #5) were obtained from Cell Signaling. anti-NUDT15 (rabbit, cat. #GTX32759, lot #822105550) was purchased from GeneTex. anti-V5 tag (mouse, clone SV5-Pk1, cat. #46-0705, lot #2735895) and anti-V5 tag (mouse, clone E10/V4RR, cat. #MA5-15253, lot #XI358694) was purchased from Invitrogen (now Thermo Fisher Scientific). anti-NUDT5 (rabbit polyclonal) was generated in-house as previously described73. anti-MTH1 (NUDT1, rabbit, cat. #NB100-109, lot #F-2) was obtained from Novus Biologicals. anti-p-Histone H3 Ser10 (rabbit, cat. #ab5176, lot #GR3396345-3), anti-β-actin (mouse, clone AC-15, cat. #ab6276, lot #0000182472), anti-α-tubulin (mouse, clone DM1A, cat. #ab7291, lot #GR3341361-15), and anti-OGG1 (rabbit recombinant, clone EPR4664(2), cat. #ab124741) were purchased from Abcam. anti-p-Histone H2A.X Ser139 (H2A.X, mouse, cloneJBW301, cat. #05-636, lot #3313712), and pan-ADP-ribose binding reagent (rabbit Fc tag, cat. #MABE1016, lot #2901597) were obtained from Millipore. anti-DHFR (rabbit, cat. #15194-1-AP, lot #00102546), anti-MTH1 (mouse, clone 2D7G4, cat. #67443-1-Ig, lot #10011993), and anti-PARP2 (rabbit, cat. #55149-1-AP, lot #00073384) were purchased from ProteinTech. Donkey anti-mouse IgG IRDye 680RD (cat. #925-68072, lot #D20803-13) and goat anti-rabbit IgG IRDye 800CW (cat. #925-32211, lot #D21109-25) were purchased from Li-Cor. anti-mCherry (rabbit, cat. # PA5-34974, lot #VB2946310D), donkey anti-mouse IgG Alexa Fluor 488 (cat. #A-21202, lot #1696430), donkey anti-mouse IgG Alexa Fluor 555 (cat. #A-31570, lot #2387458), donkey anti-rabbit IgG Alexa Fluor 568 (cat. #A-10042, lot #1020757), donkey anti-rabbit IgG Alexa Fluor 647 (cat. #A-31573, lot #2420695), goat anti-rabbit IgG Alexa Fluor 488 (cat. #A-11008, lot #913909), and donkey anti-mouse IgG Alexa Fluor 647 (cat. #A-31571, lot #1839633) were purchased from Thermo Fisher Scientific.
Validation	anti-HA probe (cat. no. sc7392) – specific to influenza hemagglutinin (HA; https://www.scbt.com/p/ha-probe-antibody-f-7); confirmed in the current manuscript using doxycycline-inducible promoters by western blot and fluorescence microscopy (Fig. 1b, Fig. 4g)<br>anti-GFP (cat. no. sc8334) – specific to green fluorescent protein (https://www.scbt.com/p/gfp-antibody-fl); confirmed in the current manuscript using doxycycline-inducible promoters by western blot and immunofluorescence microscopy (Fig. 1c, Suppl. Fig. 13)<br>anti-GFP (cat. no. sc9996) – specific to green fluorescent protein (https://www.scbt.com/p/gfp-antibody-b-2); confirmed in the current manuscript using doxycycline-inducible promoters by immunofluorescence microscopy (Suppl. Fig. 13a)<br>anti-PARP1 (cat. no. sc8007) – reactivity towards human PARP1; western blot confirmation in multiple human cell lines and by RNAi-

mediated knock-down of PARP1 (https://www.scbt.com/p/parp-1-antibody-f-2); validated using doxycycline-inducible promoters by western blot (Fig. 1c, 5b)
anti-SOD1 (cat. no. sc17767) – specific for human SOD1; confirmed by western blot in multiple human cell lines by manufacturer (https://www.scbt.com/p/sod-1-antibody-g-11) and by western blot in the current study (Fig. 1b)
anti-CHK1 (cat. no. 2360S) – reactivity towards human, mouse, rat, and monkey CHK1; confirmation by western blot in multiple cell lines and validated by multiple RNAi-mediated knock-down of CHK1 (https://www.cellsignal.com/products/primary-antibodies/chk1-2g1d5-mouse-mab/2360)
anti-p-CHK1 Ser345 (cat. no. 2341S) – reactivity towards human, mouse, rat, and monkey p-CHK1 Ser345; confirmation by western blot in multiple cell lines and known activators of CHK1 phosphorylation at Ser345 (https://www.cellsignal.com/products/primary-antibodies/phospho-chk1-ser345-antibody/2341)
anti-p-CHK1 Ser345 (cat. no. 2348S) – reactivity towards human, mouse, rat, and monkey p-CHK1 Ser345; confirmation by western blot in multiple cell lines and known activators of CHK1 phosphorylation at Ser345 but also by immunofluorescence microscopy (https://www.cellsignal.com/products/primary-antibodies/phospho-chk1-ser345-133d3-rabbit-mab/2348)
anti-p-Histone H2A.X Ser139 (H2A.X, cat. no. 2577S) – reactivity towards human, mouse, rat, and monkey p-H2A.X Ser139; confirmation by western blot and known activators of CHK1 phosphorylation at Ser345 but also by immunofluorescence microscopy (https://www.cellsignal.com/products/primary-antibodies/phospho-histone-h2a-x-ser139-antibody/2577)
anti-vinculin (cat. no. 4650S) – reactivity towards human, mouse, rat, monkey, and dog vinculin; confirmation by western blot in multiple cell lines (https://www.cellsignal.com/products/primary-antibodies/vinculin-antibody/4650)
anti-NUDT15 (cat. no. GTX32759) – reactivity towards human NUDT15; confirmed applications for western blotting and immunohistochemistry (https://www.genetex.com/Product/Detail/NUDT15-antibody/GTX32759)
anti-V5 tag (cat. no. 46-0705) – specific to the V5 epitope; confirmed applications for western blotting and immunofluorescence (https://www.thermofisher.com/antibody/product/V5-Tag-Antibody-clone-SV5-Pk1-Monoclonal/R960-25).
anti-V5 tag (cat. no. MA5-15253) – specific to the V5 epitope; confirmed applications for western blotting (this study; Fig. 1a), immunofluorescence, ELISA, immunoprecipitation (https://www.thermofisher.com/antibody/product/V5-Tag-Antibody-clone-E10-V4RR-Monoclonal/MA5-15253), and flow cytometry (this study; Suppl. Fig. 6e).
anti-NUDT5 (rabbit polyclonal) – validated previously by RNAi-mediated depletion in multiple human cell lines by western blotting in Page & Valerie, et al., Nature Comm, 2018 (https://doi.org/10.1038/s41467-017-02293-7)
anti-MTH1 (NUDT1, cat. no. NB100-109) – reactivity towards human (manufacturer) and rat MTH1 (PMID: 12706856); confirmation of specificity by western blot in MTH1 knock-out in human cells (https://www.bio-techne.com/p/antibodies/mth1-antibody_nb100-109)
anti-p-Histone H3 Ser10 (cat. no. ab5176) – reactivity towards human phosphorylated histone H3 Ser10; specificity confirmed by known chemical inducers of histone H3 Ser10 phosphorylation and specific peptide competition (https://www.abcam.com/en-us/products/primary-antibodies/histone-h3-phospho-s10-antibody-ab5176)
anti-β-actin (cat. no. ab6276) – reactivity towards mouse, rat, cow, dog, human, African green monkey, Chinese hamster beta actin; confirmed specificity by beta actin knock-out in human cells with western blotting (https://www.abcam.com/en-us/products/primary-antibodies/beta-actin-antibody-ac-15-ab6276).
anti-α-tubulin (cat. no. ab7291) – reactivity towards rat, human, and mouse samples; confirmation by immunohistochemistry, flow cytometry, western blot, and immunofluorescence (https://www.abcam.com/en-us/products/primary-antibodies/alpha-tubulin-antibody-dm1a-loading-control-ab7291).
anti-OGG1 (cat. no. ab124741) – reactivity towards human OGG1; confirmation by knockout; validation by western blot (this study) and immunohistochemistry (manufacturer)
anti-p-Histone H2A.X Ser139 (H2A.X, cat. no. 05-636) – reactivity towards vertebrate H2A.X phosphorylated on Ser139; specificity is shown with UV-treated human cells and breast cancer tissue (https://www.merckmillipore.com/SE/en/product/Anti-phospho-Histone-H2A.X-Ser139-Antibody-clone-JBW301,MM_NF-05-636?ReferrerURL=https%3A%2F%2Fwww.google.com%2F)
pan-ADP-ribose binding reagent (rabbit Fc tag, cat. no. MABE1016) – reactivity towards human and mouse mono- and poly-ADP-ribosylated proteins by western blotting (https://www.sigmaaldrich.com/SE/en/product/mm/mabe1016) but predicted to react with all species
anti-DHFR (15194-1-AP) – reactivity towards human, mouse, rat, zebrafish; confirmed with western blot, immunoprecipitation, immunohistochemistry, immunofluorescence, and ELISA (https://www.ptglab.com/products/DHFR-Antibody-15194-1-AP.htm); also confirmed in this study by western blot using doxycycline-inducible promoters (Suppl. Fig. 3a).
anti-MTH1 (cat. no. 67443-1-Ig) – reactivity towards human, mouse; confirmed with western blot, immunohistochemistry, ELISA (https://www.ptglab.com/products/MTH1-Antibody-67443-1-Ig.htm); confirmed by western blot in this study with doxycycline-inducible promoters (Suppl. Fig. 6b).
anti-PARP2 (cat. no. 55149-1-AP) – reactivity towards human; confirmed with western blot, immunofluorescence, ELISA (https://www.ptglab.com/products/PARP2-Antibody-55149-1-AP.htm); confirmed in this study by western blot with endogenous and exogenous protein (Fig. 2g).
anti-mCherry (cat. no. PA5-34974) – reactivity towards mCherry tag (https://www.thermofisher.com/antibody/product/mCherry-Antibody-Polyclonal/PA5-34974); specificity confirmed in the current manuscript with mCherry-expressing cells by western blot (Suppl. Fig. 17e).

Eukaryotic cell lines

Policy information about cell lines and Sex and Gender in Research

Cell line source(s)	U-2 OS osteosarcoma (HTB-96), HEK293T embryonic kidney epithelial (293T; CRL-3216), and KG-1 acute myelogenous leukemia (AML; CCL-246) cells were obtained from the American Type Culture Collection (ATCC, Manassass, VA, USA). HCT116 and HCT116 3-6 colon carcinoma cells were originally obtained from Dr. Bert Vogelstein (Johns Hopkins University).
Authentication	ATCC-derived cultures are authenticated by morphological profiling, karyotyping, and PCR-based methodologies (STR profiling). No further authentication was performed.
Mycoplasma contamination	Cell lines were routinely screened for mycoplasma using the MycoAlert kit (Lonza Bioscience) and tested negative.

Commonly misidentified lines (See ICLAC register)	None of the cell lines were listed as misidentified on ICLAC or known to be cross-contaminated.

Animals and other research organisms

Policy information about studies involving animals; ARRIVE guidelines recommended for reporting animal research, and Sex and Gender in Research

Laboratory animals	BALB/cAnNCrl nude mice (strain code: 194 [homozygous], Charles River Labs); 6–8 weeks old
Wild animals	The study did not involve wild animals.
Reporting on sex	Sex-based analysis was not performed in the current study. The findings presented are gender agnostic.
Field-collected samples	The study did not involve samples collected from the field.
Ethics oversight	All work was performed in accordance with EU (European Union) and Swedish Ethical Review Authority regulations for animal experimentation under Karolinska Institutet permit no. 5718-2019. Tumors did not exceed 1000 mm3 in size in this study, as mandated by the Swedish Central Animal Research Ethics Committee (Centrala Djurförsöksetiska Nämnden).

Note that full information on the approval of the study protocol must also be provided in the manuscript.

Plants

Seed stocks	*Report on the source of all seed stocks or other plant material used. If applicable, state the seed stock centre and catalogue number. If plant specimens were collected from the field, describe the collection location, date and sampling procedures.*
Novel plant genotypes	*Describe the methods by which all novel plant genotypes were produced. This includes those generated by transgenic approaches, gene editing, chemical/radiation-based mutagenesis and hybridization. For transgenic lines, describe the transformation method, the number of independent lines analyzed and the generation upon which experiments were performed. For gene-edited lines, describe the editor used, the endogenous sequence targeted for editing, the targeting guide RNA sequence (if applicable) and how the editor was applied.*
Authentication	*Describe any authentication procedures for each seed stock used or novel genotype generated. Describe any experiments used to assess the effect of a mutation and, where applicable, how potential secondary effects (e.g. second site T-DNA insertions, mosiacism, off-target gene editing) were examined.*

Flow Cytometry

Plots

Confirm that:

- [x] The axis labels state the marker and fluorochrome used (e.g. CD4-FITC).
- [x] The axis scales are clearly visible. Include numbers along axes only for bottom left plot of group (a 'group' is an analysis of identical markers).
- [x] All plots are contour plots with outliers or pseudocolor plots.
- [x] A numerical value for number of cells or percentage (with statistics) is provided.

Methodology

Sample preparation	Cell lines used for flow cytometry were U-2 OS or HCT116 cell lines previously transduced with recombinant CeTEAM biosensors.<br><br>For MTH1 studies, 400,000 U-2 OS V5-MTH1 G48E clone #6 cells were plated in T25 flasks in the presence of 1 µg/mL DOX on day 0. The following morning (day 1), the cells were then treated with DMSO (0.01% v/v final concentration) or the indicated concentration of MTH1i for 24 hours. On the morning of day 2, the cells were harvested by trypsinization and pooling of culture medium, as well as PBS washes (to ensure collection of dead and mitotic cells). Following a wash with PBS, the cells were fixed with 4% PFA in PBS for 15 minutes, washed once with 1% BSA/PBS, then permeabilized with saponin buffer (0.1% saponin in 1% BSA/PBS) on ice for 30 minutes. The cells were then stained with anti-V5 (mouse monoclonal, 1:300) and anti-p-HH3 Ser10 (rabbit polyclonal, 1:500) antibodies diluted in saponin buffer overnight at 4ºC. Next, the cells were washed twice with saponin buffer prior to incubation with donkey anti-rabbit Alexa Fluor 647 and donkey anti-mouse Alexa Fluor 488 antibodies (1:1000 in saponin buffer) for 30 minutes at 37ºC. Following two additional washes with saponin buffer, the cells were incubated with 0.1 mg/mL RNase A (Thermo Fisher Scientific) and 10 µg/mL Hoechst 33342 for 15 minutes at room temperature in 1% BSA/PBS. Control V5-MTH1 G48E cells were also used for singlet antibody controls (one for V5 and one for p-HH3 Ser10).<br><br>PARP1 experiments were performed with live U-2 OS pINDUCER20-PARP1 L713F-GFP #5/pLenti CMV Blast-mCherry or HCT116 pLenti CMV Blast-PARP1 L713F-GFP/pLenti CMV Blast-mCherry cells. Briefly, 200,000 cells were plated in T25 flasks (in the presence of 1 µg/mL DOX for U-2 OS cells). The following day, varying concentrations of veliparib/niraparib or an

equivalent volume of DMSO was added to the cells prior to harvesting 24 hours later. Trypsinized cells were quenched with complete medium, pelleted at 400 x g for 5 minutes, washed by resuspending in sterile PBS/10% FBS, centrifuged again, and then transferred to 5 mL flow cytometry tubes via a 40 µm strainer cap (BD Falcon) in 500 µL sterile PBS/5% FBS.

Dissociation of excised subcutaneous HCT116 L713F-GFP/mCherry tumors was performed similarly to previously reported methods. Briefly, approximately 60-80 mm3 of tumor tissue was finely minced with a sterile scalpel and dissociated for 1 hour at 37°C with shaking in 9 mL of an enzyme solution containing 1 mg/mL collagenase D (Sigma) and 100 ng/mL DNAse I (≥40 U/mL final; Sigma) in McCoy's 5a GlutaMAX without additives. For the final 5 minutes of the incubation period, 1 mL TrypLE Express was added to each tube. Digestive enzymes were deactivated by the addition of 3 mL McCoy's 5a medium containing 10% FBS and the cell suspension was sieved through a 40 µm strainer (Corning), followed by an additional rinse with 2 mL McCoy's/10% FBS. The cells were pelleted by centrifugation at 400 x g for 5 minutes and washed by resuspension in sterile PBS/10% FBS. After centrifugation, the cell pellet was resuspended in 1 mL sterile PBS/5% FBS and transferred to a 5 mL flow cytometry tube via a 40 µm strainer cap (BD Falcon). Viability of the final samples was between 30-35% by trypan blue exclusion.

Instrument	Bectin Dickenson BD LSR Fortessa (MTH1 experiments); Bectin Dickenson BD Accuri C6 Plus (PARP1 experiments)
Software	Events were acquired using BD FACSDiva software (Version 8.0.1) (for MTH1 experiments). Events were acquired and analyzed with BD Accuri C6 Plus Software [for PARP1 experiments]. Additional analyses, including export of raw, per-event values, were performed with FlowJo v10.7.1 (Bectin Dickenson).
Cell population abundance	All analysis was of bulk cell populations from cultured cell lines. Viability of cells from dissociated HCT116 xenografts were assessed to be 30-35% by trypan blue exclusion and live tumor cells were identified by mCherry fluorescence.
Gating strategy	FSC/SSC gates were determined by the location/clustering of the majority of cells to determine "live" cells. This was straightforward with isolated cell lines. Single cells (singlets) were determined by FSC-Area or FSC-Width plots. "V5 high" cells were arbitrarily determined as cells equal to or brighter than the equivalent of the top 2% of DMSO control cells. "pHH3+" cells are a small subset of cells (mitotic fraction) that clearly separate from pHH3– cells.

☒ Tick this box to confirm that a figure exemplifying the gating strategy is provided in the Supplementary Information.
